# Biodegradable microplastics influence on organic component microbial transformation during sludge composting

**DOI:** 10.3389/fmicb.2026.1830632

**Published:** 2026-05-12

**Authors:** Zixuan Wang, Yuewei Du, Yunfei Gao, Xinyu Zhao, Ting Yang

**Affiliations:** 1 College of Life and Environmental Sciences, Minzu University of China, Beijing, China; 2 Innovation Base of Groundwater and Environmental System Engineering, Chinese Research Academy of Environmental Sciences, Beijing, China

**Keywords:** biodegradable microplastics, high-throughput sequencing, microbial community, organic components, sludge composting

## Abstract

Sludge-based composting offers a promising pathway for sustainable resource recovery and pollution mitigation; however, the biodegradation mechanisms of biodegradable microplastics (MPs) during this process and their interactions with organic matter transformation have not yet been investigated. This study investigated the biodegradation response of polylactic acid (PLA) and the coupled dynamics of microbial communities and organic matter transformation during a 33-day sludge composting process at 55 °C and 70 °C. Scanning electron microscopy (SEM) revealed that thermophilic composting (70 °C) induced severe structural degradation of PLA-MPs, characterized by extensive void formation, surface wrinkling, and fragmentation. Compared to the mesophilic control, thermophilic composting significantly enhanced the transformation and turnover of key organic components, including amino acids (AAs), reducing sugars (RSs), polysaccharides, and polyphenols (PPs). Bacterial communities were predominantly composed of thermophilic Firmicutes (74.6–98.2%), with significant contributions from Actinobacteria, Chloroflexi, and Bacteroidetes, while fungal communities were dominated by Ascomycota and Basidiomycota. Although the addition of MPs reduced overall microbial richness and diversity, high-temperature conditions selectively enriched key organic matter-degrading taxa, suggesting a functional trade-off where thermal pressure favors specialized degradative capacity over generic community complexity. Co-occurrence network analysis revealed that high-temperature composting combined enhanced microbial functional connectivity and metabolic redundancy for both MPs and organic component transformation, promoting the proliferation of polysaccharides and lignocellulose-decomposing bacteria and fungi. These findings provide mechanistic insights into biodegradable MP degradation during thermophilic composting and establish a theoretical foundation for designing efficient MP remediation strategies in sludge treatment systems. Future studies are warranted to evaluate these findings under field-scale composting conditions and explore the integration of microbial inoculants to optimize the removal efficiency of biodegradable MPs.

## Graphical abstract

**Figure:**
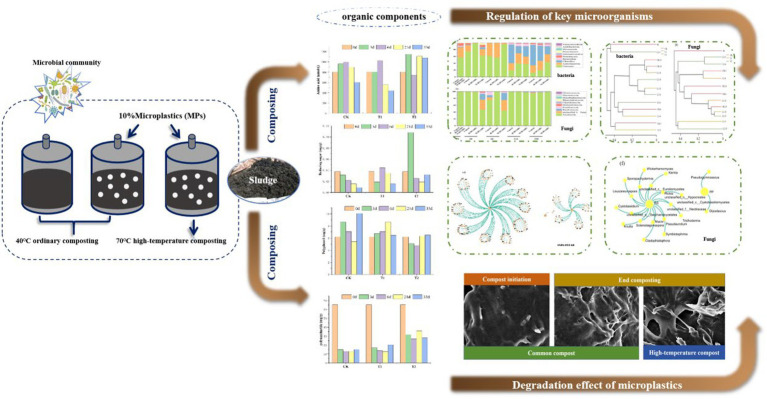

## Highlights

- High-temperature composting significantly promoted MPs degradation.
- MPs promoted microbial conversion of organic components.
- MPs showed stronger inhibition on fungi than bacteria.
- High-temperature MP degradation had stronger ecological effects.

## 1 Introduction

Microplastics (MPs) are defined as plastic particles with a size range of less than 5 mm, originating from two primary pathways: (1) primary MPs, which are intentionally manufactured at small sizes, and (2) secondary MPs, which are generated through the physical, chemical, and biological fragmentation of larger plastic debris (Chen Z. et al., 2020; Wang et al., 2021). Due to their small size, large specific surface area, and good dispersibility and hydrophobicity, MPs can easily adsorb organic chemical pollutants, heavy metals, pathogens, and microorganisms, thereby indirectly threatening the health of marine organisms and humans (Cao et al., 2021; Lambert et al., 2024; Wang J. et al., 2024). As a new type of pollutant, MPs have been increasingly detected in sludge (Kong et al., 2024; Ma et al., 2024). Sludge is widely utilized as a soil amendment and fertilizer due to its high organic matter and nutrient content. However, its application has emerged as a significant pathway for MPs to enter terrestrial and aquatic ecosystems, raising concerns about environmental contamination (Chand et al., 2024). In addition, co-composting technology has been demonstrated as an effective approach for reducing MP abundance in sludge (Corradini et al., 2019; Sun et al., 2023; Wang Y. et al., 2024), and composting can further facilitate the degradation of biodegradable MPs and decrease their residual concentration (Sun et al., 2021). Furthermore, temperature is a critical factor regulating the biotransformation of biodegradable MPs during composting (Xi et al., 2022). Elevated temperatures can accelerate the cleavage of C–C bonds in polymer chains, facilitating subsequent microbial degradation (Sun et al., 2021). Moreover, temperature shifts drive temporal succession in microbial community composition, which, in turn, influences the generation and transformation of organic intermediates during the composting process. However, the coupled dynamics among temperature-driven microbial community shifts, organic matter transformation, and biodegradable MP degradation remain poorly understood.

Microorganisms decompose organic matter and release a large amount of heat, making the surface of biodegradable MPs rough and the structure easy to change (Li et al., 2022). Meanwhile, microorganisms play a key role as decomposers in farmland soils, compost systems, and marine ecosystems (Li et al., 2024; Peixoto et al., 2017; Wang Y. F. et al., 2024; Zhang et al., 2024), thereby promoting the degradation of MPs during sludge composting. Chen X. M. et al. (2020) reported that thermophilic bacteria in compost can effectively degrade 43.7% of MPs in sewage sludge. Microorganisms play an important role during the composting process (You et al., 2024), and the abundance, community composition, diversity, and community structure of microorganisms vary under different temperatures (Liu et al., 2023; Liu N. Y. et al., 2024). With the help of microorganisms, organic components [amino acids (AAs), reducing sugars (RSs), polyphenols (PPs), and polysaccharides] are first produced through their own metabolic activities and the decomposition of organic substrates. These small-molecule compounds are closely associated with microbial community composition and diversity (Xing et al., 2023; Yu et al., 2022), as microbial communities play a central role in the transformation of organic components (Huang et al., 2019). Amino acids and polyphenols may enhance the decomposition ability of MPs by stimulating the metabolic pathway of microorganisms (Chen et al., 2024; Li et al., 2023). This is primarily because organic components such as amino acids and reducing sugars provide nutrients as energy and carbon sources for microorganisms to promote their reproduction and metabolism (Chang et al., 2023). Organic components dynamically influence MP degradation through multiple pathways. Polyphenols enhance microbial metabolic capacity for MP degradation by stimulating specific enzymatic pathways (Xu et al., 2022), while other decomposition intermediates (e.g., humic substances and organic acids) may form complexes with MPs, potentially modifying their accessibility and degradation rates (Lin et al., 2024). These dual effects underscore the importance of understanding the dynamics of organic components in regulating MP degradation during composting. However, how thermophilic conditions simultaneously reshape microbial community structure and alter organic component composition—and how these coupled changes collectively influence biodegradable MP degradation—remains unexplored.

Therefore, this study aimed to investigate: (1) the biodegradation kinetics of polylactic acid (PLA)-MPs under thermophilic conditions, (2) the temperature-driven succession of microbial communities, (3) the transformation dynamics of organic components, and (4) the functional coupling between microbial assemblages and organic matter in regulating MP degradation efficiency.

## 2 Materials and methods

### 2.1 Composting materials and experimental design

The sludge used in this experiment was collected from the secondary sedimentation tank of a sewage treatment plant in Beijing. The C/N ratio was adjusted to 25:1 using straw and sawdust. PLA-MPs were obtained from a plastic material supplier in Shanghai. In the high-temperature treatment group, quicklime was thoroughly mixed with PLA-MPs buried in compost nylon mesh bags (Ye et al., 2023). According to the amount of microplastics added and the composting temperature, three treatment groups were established: (1) ordinary sludge compost without microplastics at 55 °C (CK), (2) ordinary sludge compost with 10% microplastics at 55 °C (L1), and (3) high-temperature sludge compost with 10% microplastics at 70 °C (L2). The 10% (w/w) PLA-MP addition ratio was used as an elevated dosage to strengthen biological signals and better characterize the responses of microbial communities and organic components over the 33-day composting period. Although this ratio exceeded the MP concentrations typically reported in real sewage sludge (El Hayany et al., 2022), such high-dosage designs are commonly used in laboratory-scale studies to reveal ecological effects that are difficult to detect at lower concentrations. Each treatment was conducted in triplicate with a composting cycle of 33 days. The temperature changes during sludge composting are shown in Supplementary Figure S1. A total of five sampling points were set at days 0, 3, 6, 21, and 33, with three replicates for each. Approximately 60 g of samples were ground to 0.15 mm after natural air drying and used for physicochemical analysis. The remaining wet samples were stored in a refrigerator at −20 °C. Fresh compost samples were selected for high-throughput sequencing and dynamic analysis of the microbial community.

### 2.2 Extraction and measurement of organic components

3,5-Dinitrosalicylic acid was used to determine reducing sugars (RSs) at OD_540_ nm (Cao et al., 2013). AAs were determined using the ninhydrin chromogenic assay at OD_570_ nm (Wu et al., 2020). Polyphenolic (PP) compounds were determined by extracting 1 g of the sample three times with 80% methanol, followed by evaporation, and the concentration was determined using an Agilent 1260 high-performance liquid chromatography system (Agilent, United States) (Rigane et al., 2015). Polysaccharide (SS) determination was carried out according to Cao’s method (Cao et al., 2013). The detection limits were 0.017 mg/g for polysaccharides, 0.078 mg/g for polyphenols, 0.017 mg/g for reducing sugars, and 0.091 μmol/g for amino acids.

### 2.3 16S rRNA high-throughput sequencing

Microbial community composition was analyzed using 16S rRNA gene sequencing for bacteria and internal transcribed spacer (ITS) sequencing for fungi. Genomic DNA was extracted from sludge samples stored at −80 °C. The bacterial 16S rRNA gene V3–V4 region was amplified using primers 319F (5′-ACTCCTACGGGAGGCAGCAG-3′) and 806R (5′-GGACTACHVGGGTWTCTAAT-3′). Fungal ITS regions were amplified using primers ITS3-F (5′-GCATCGATGAAGAACGCAGC-3′) and ITS4-R (5′-TCCTCCGCTTATTGATATGC-3′) (Chen et al., 2023; Guo et al., 2024). PCR amplicons were purified using Axygen magnetic beads and quantified. The amplicon libraries were sequenced using the Illumina MiSeq platform (2 × 300 bp paired-end reads, Illumina, United States). Raw sequences were processed using the QIIME2 pipeline, where OTU clustering (97% similarity threshold) or ASV inference was performed, followed by taxonomic classification against the SILVA v138.1 (bacterial) and UNITE v10.0 (fungal) databases (Möhlmann et al., 2020).

### 2.4 Statistical analysis

OriginPro 2024 (OriginLab, United States) was used to visualize the contents of organic components (amino acids, reducing sugars, polyphenols, and polysaccharides) and the relative abundance of microorganisms. SPSS statistical software was used to perform Pearson correlation and significance analyses of microbial abundance and organic components. Cytoscape (V3.10.4, United States) was used to map the network of organic components and key microorganisms (Chen X. M. et al., 2020). The Shannon index and non-metric multidimensional scaling (NMDS) analysis were performed using Canoco for Windows (Version 5.0, Netherlands) to characterize dynamic changes in microbial community diversity and structure. Correlation heat maps between organic components and physicochemical indices were generated using R (v4.2.1) software. IBM SPSS Amos 26.0 software (IBM, United States) was used to establish a structural equation model to evaluate the interactions between environmental factors and microbial communities.

## 3 Results

### 3.1 Impact of temperature and PLA-MP addition on organic components

**Figure 1:**
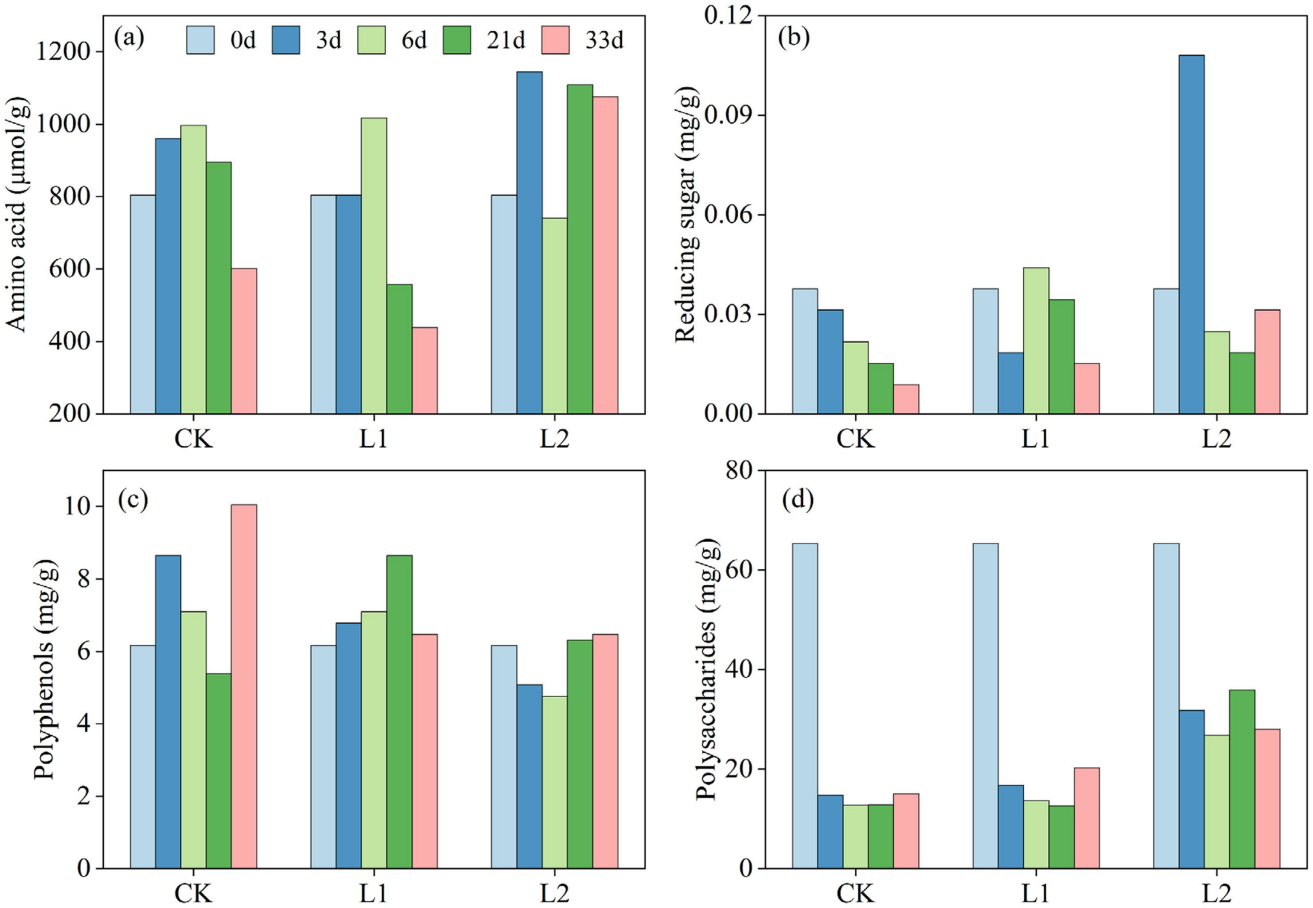
Changes in the contents of organic components [**(a)** Amino acids (AAs), **(b)** reducing sugars (RSs), **(c)** polyphenols (PPs), and **(d)** polysaccharides (SS)] uring sludge composting with MPs under different temperature conditions. CK: Ordinary sludge compost without microplastics at 55 °C; L1: Ordinary sludge compost with 10% microplastics added at 55 °C; L2: High-temperature sludge compost with 10% microplastics added at 70 °C.

Organic matter degradation occurs simultaneously during composting (Gao et al., 2024). Polyphenols (PP), amino acids (AA), reducing sugars (RS), and polysaccharides (SS) in organic components are also important precursors of humic acid (Huang et al., 2019). To investigate the impacts of PLA-MPs on organic components during composting at different temperatures, the dynamic changes in PP, AA, RS, and SS in the CK, L1, and L2 groups were studied over a 33-day composting period (Figures 1a–d). There were significant differences in PP content during the later stages of composting. At the end of composting, PP content in groups L1 and L2 was significantly lower than that in the CK group, with the L2 group showing the lowest content. For AA, the CK, L1, and L2 groups showed an upward trend during the early and middle stages, with increases of 24.3 and 26.3% in the L1 and L2 groups, respectively. AA content in the L1 group was significantly lower than that in the control group in the later stage. RS content in the CK group showed a continuous decreasing trend, with a decrease range of 2.3–2.9%. RS content in the L1 group fluctuated within a certain range. The L2 group showed enhanced accumulation of RS content under the combined action of high temperature and MPs. Polysaccharide content in the three treatments showed a significant decreasing trend during the first 3 days, while it remained basically unchanged in the later stage of composting. After composting, the polysaccharide concentration in the L1 group was slightly higher than that in the CK group. At the end of composting, polysaccharide content under high-temperature composting was significantly higher than that under ordinary composting.

### 3.2 Effect of high-temperature composting with PLA-MPs on microbial communities

**Figure 2:**
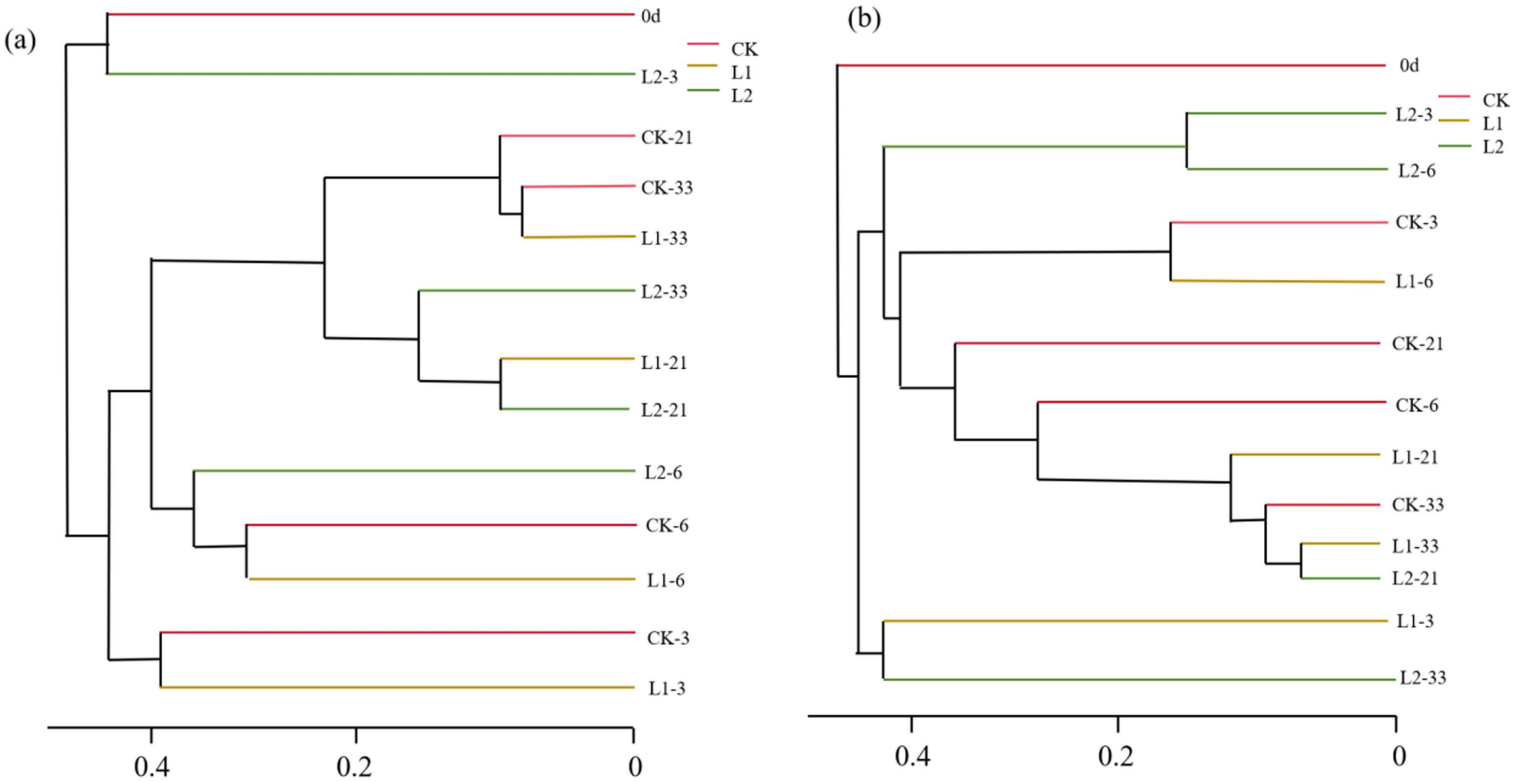
Cluster analysis of bacteria **(a)** and fungi **(b)** in different composting processes.

Bacterial community composition on the third and sixth days was similar in the CK and L1 groups, but significant differences were observed on day 21 (Figure 2). During the high-temperature composting process, bacterial community composition showed significant differences at different composting stages compared to the control group and ordinary composting. As shown in Figure 2, fungal community composition in the CK and L1 groups differed significantly at most composting stages, except on day 33. Similarly, fungal community composition in the high-temperature composting group was more distinct from that in the CK and L1 groups.

**Figure 3:**
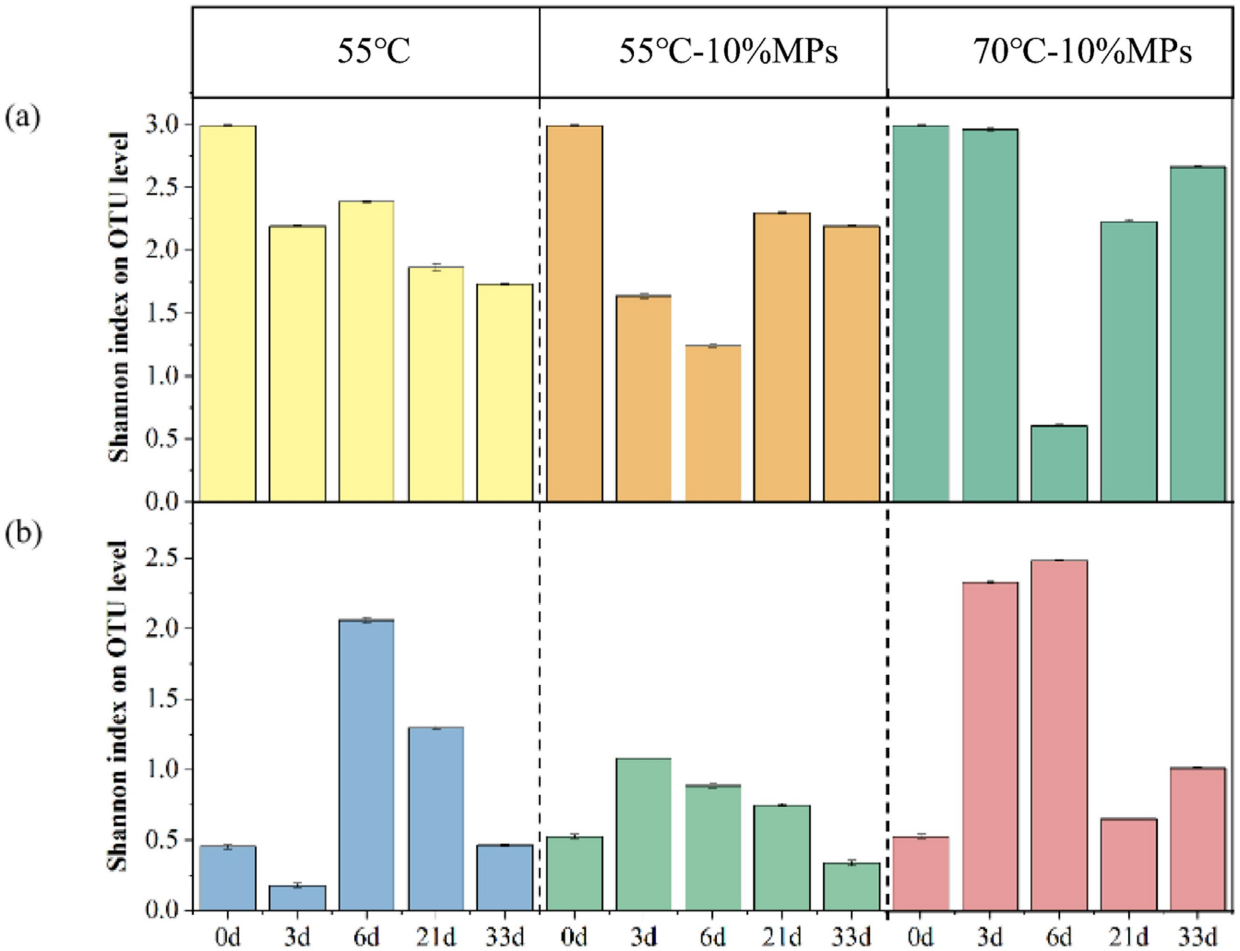
Shannon index of bacteria **(a)** and fungi **(b)** during sludge composting with MPs under different temperature conditions. CK: Ordinary sludge compost without microplastics at 55 °C; L1: Ordinary sludge compost with 10% microplastics added at 55 °C; L2: High-temperature sludge compost with 10% microplastics added at 70 °C.

NMDS and the Shannon index were used to evaluate the abundance and diversity of microbial communities (Duan et al., 2022). As shown in Figure 3, compared to the CK group, the Shannon indices of fungi and bacteria in the L1 group were significantly reduced. In the L2 group, the Shannon indices of fungi and bacteria were increased.

**Figure 4:**
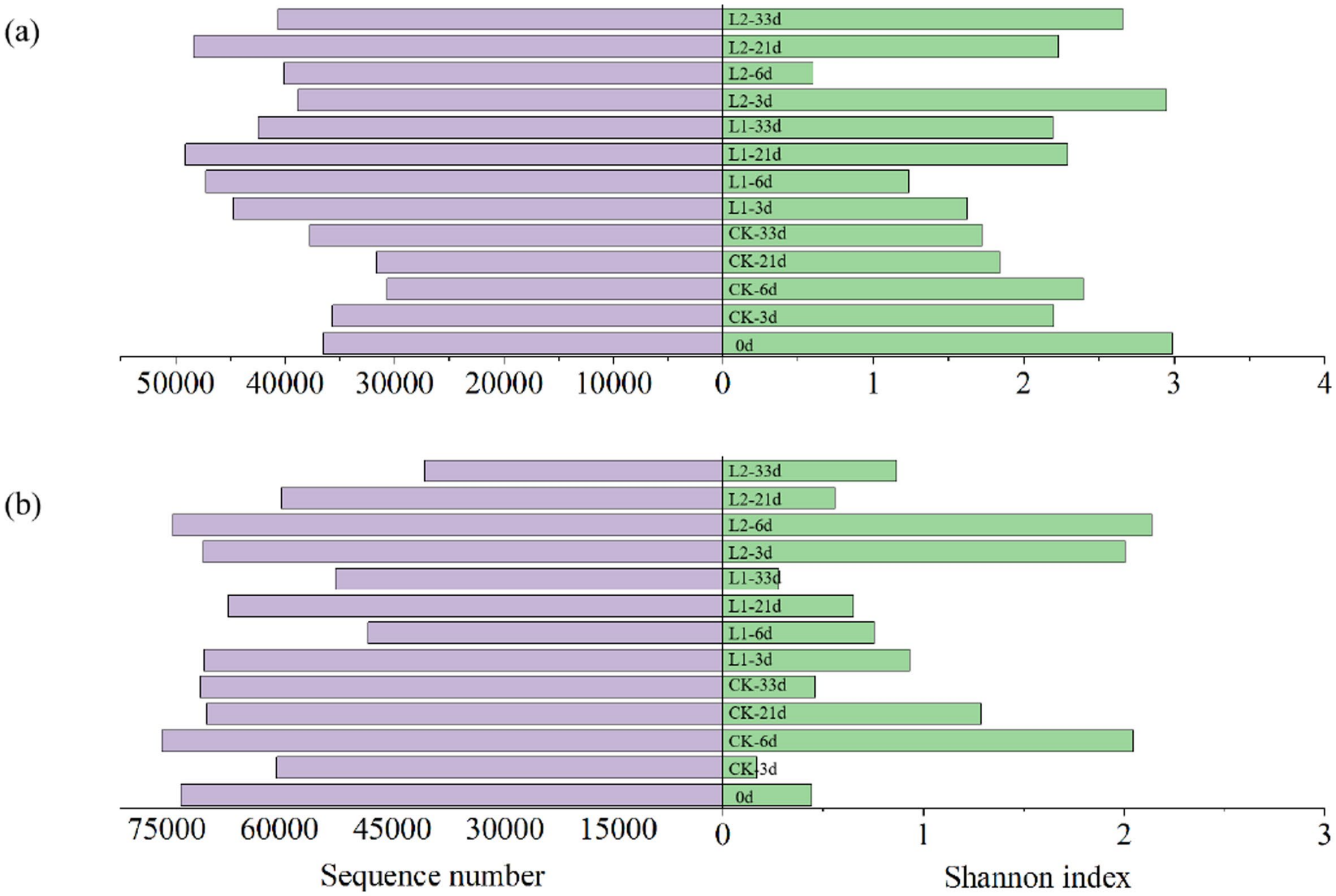
Shannon index and total abundance of bacteria **(a)** and fungi **(b)** during sludge composting with MPs under different temperature conditions. CK: Ordinary sludge compost without microplastics at 55 °C; L1: Ordinary sludge compost with 10% microplastics added at 55 °C; L2: High-temperature sludge compost with 10% microplastics added at 70 °C.

As shown in Figure 4a, the number of bacterial sequences in the L1 group was higher than that in the CK group, but the Shannon index was lower than that in the CK group. The number of bacterial sequences in the L2 group was also higher than that in the CK group, and the Shannon index was slightly higher than that in the CK group. In addition, the number of bacterial sequences in the L2 group was slightly lower than that in the L1 group, and the Shannon index was slightly higher. As shown in Figure 4b, the number of fungal sequences in the L1 group was lower than that in the CK group, and the Shannon index of fungi was also lower than that in the CK group. The overall abundance of fungi decreased in the L2 group, but their diversity increased.

**Figure 5:**
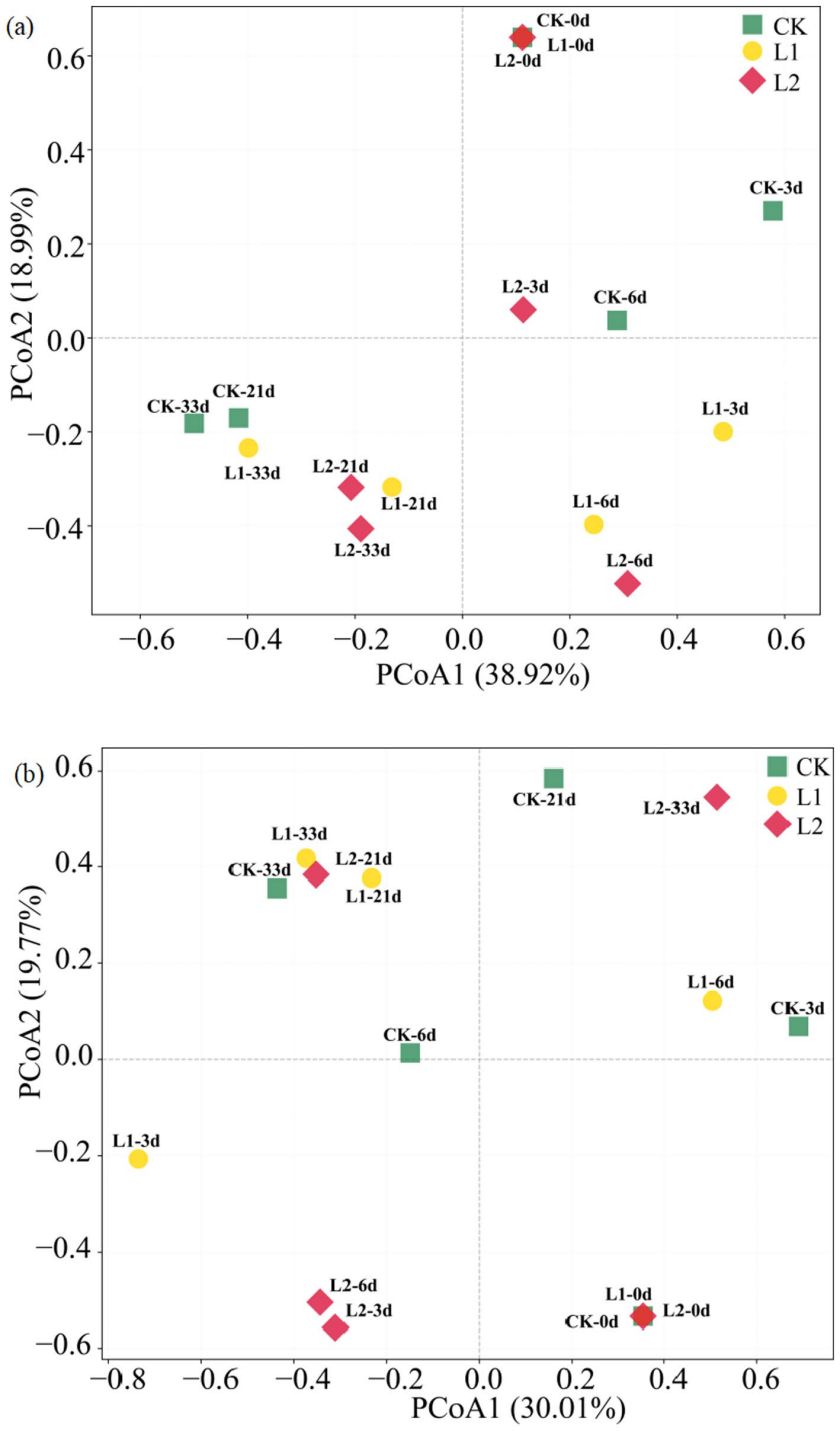
NMDS analysis based on bacterial **(a)** and fungal **(b)** microbial communities. CK: Ordinary sludge compost without microplastics at 55 °C; L1: Ordinary sludge compost with 10% microplastics added at 55 °C; L2: High-temperature sludge compost with 10% microplastics added at 70 °C.

Bacterial communities during composting in the different treatment groups (Figures 5a,b) could be divided into two categories. The samples clustered together during the cooling and maturation periods (21 d and 33 d) of composting. The succession trend in the L1 group was similar to that in the CK group, whereas the other category primarily consisted of samples collected on days 3 and 6. The clustering of 21-d and 33-d samples indicated stabilization of the bacterial community. Figure 5b shows the NMDS diagram of the distribution of fungal communities. Unlike the distribution of bacterial communities, most fungal communities were widely dispersed across different composting stages. In the composting process, the distribution of fungal communities was more dispersed, and the difference was significant throughout the entire composting period. For fungi, the community structure of the L2 group was significantly different from that of the CK and L1 groups.

### 3.3 Changes in microbial community composition during composting with PLA-MPs

**Figure 6:**
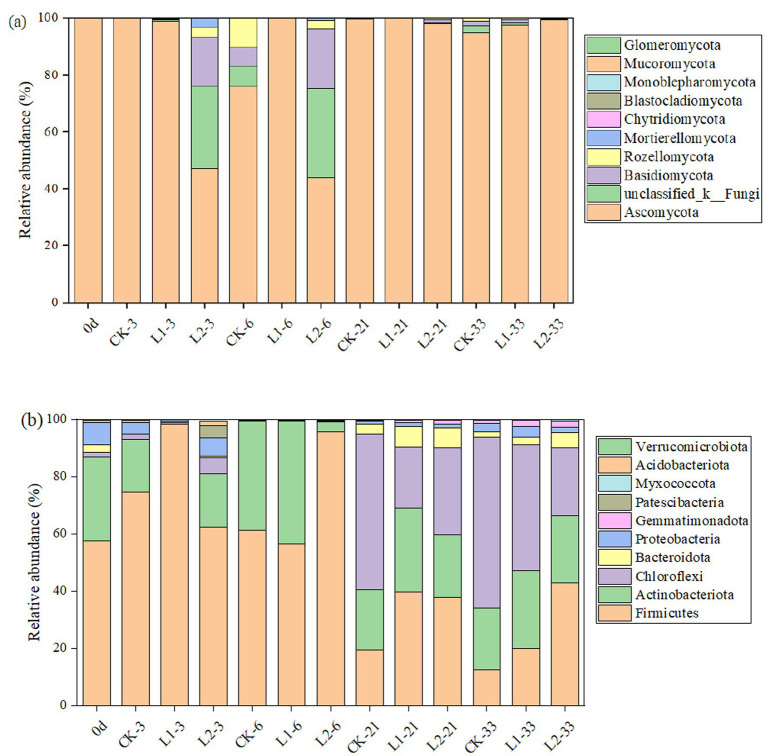
Top 10 phyla of bacteria **(a)** and fungi **(b)** based on relative abundance. CK: Ordinary sludge compost without microplastics at 55 °C; L1: Ordinary sludge compost with 10% microplastics added at 55 °C; L2: High-temperature sludge compost with 10% microplastics added at 70 °C.

To investigate the effect of MP addition under high-temperature conditions on the relative abundance of bacteria and fungi during composting, this study selected the top 10 bacterial and fungal phyla for analysis (Figure 6). The main bacterial phyla were Firmicutes, Actinobacteria, Chloroflexi, Proteobacteria, and Bacteroidetes (Figure 6a). The Firmicutes phylum in both the CK and L1 groups showed a trend of first increasing and then decreasing, reaching a maximum abundance of 74.6 and 98.2% at 3 d. The Firmicutes phylum in the L2 group was significantly higher than that in the CK group, and the average level in the later stage of composting was also much higher than that in the CK and L1 groups. In each treatment group, the abundance of Actinobacteria fluctuated within a certain range. Among them, the relative abundance in the L1 group was only 0.71% at 3 d. As composting progressed, the relative abundance of Actinobacteria gradually returned to normal levels. L2 delayed the response of microorganisms to MPs under high-temperature conditions. Both Chloroflexi and Bacteroidetes appeared in the middle and late stages of composting. The relative abundance of Chloroflexi in the CK, L1, and L2 groups was 59.7, 44.0, and 23.8%, respectively. The relative abundance of Bacteroidetes in the CK, L1, and L2 groups was 1.96, 2.6, and 5.35%, respectively, after composting. Ascomycota dominated the fungal community (Figure 6b), followed by Basidiomycota, unclassified_k__Fungi, and Rozellomycota. The proportion of Basidiomycota, unclassified_k__Fungi, and Rozellomycota increased in the L2 group.

**Figure 7:**
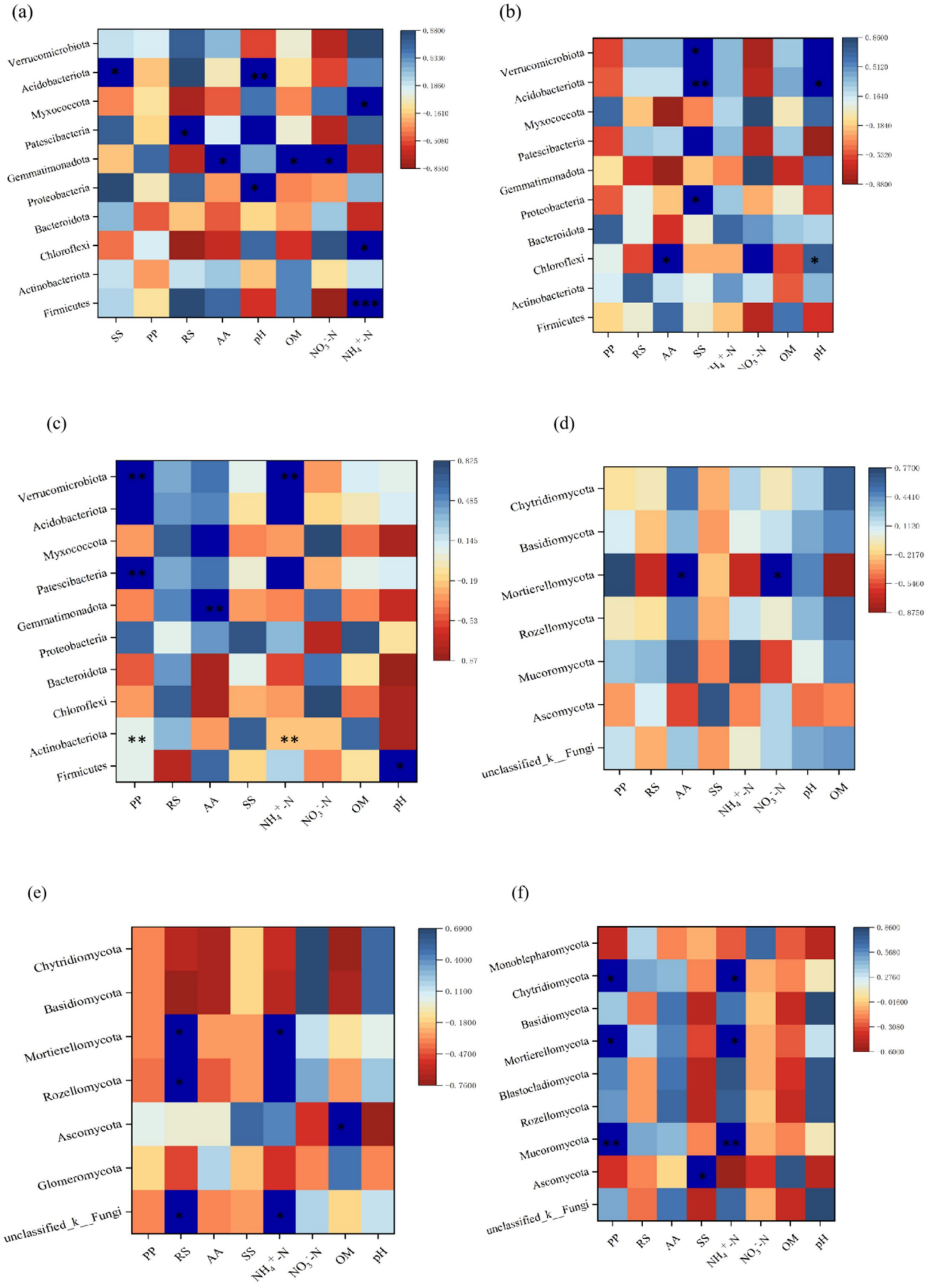
Heat maps showing correlations between bacteria (**a**-CK, **b**-L1, **c**-L2) and fungi (**d**-CK, **e**-L1, **f**-L2) at the phylum level, organic components, and physicochemical factors during sludge composting with MPs under different temperature conditions. CK: Ordinary sludge compost without microplastics at 55 °C; L1: Ordinary sludge compost with 10% microplastics added at 55 °C; L2: High-temperature sludge compost with 10% microplastics added at 70 °C; *p < 0.05, **p < 0.01, ***p < 0.001.

The relationships between microbial communities and environmental factors under different composting treatments were further analyzed. As shown in Figures 7a–f, the overall correlation patterns among the treatment groups were similar, although the strength of the correlations differed.

### 3.4 Co-occurrence network analysis of functional microbial communities

**Figure 8:**
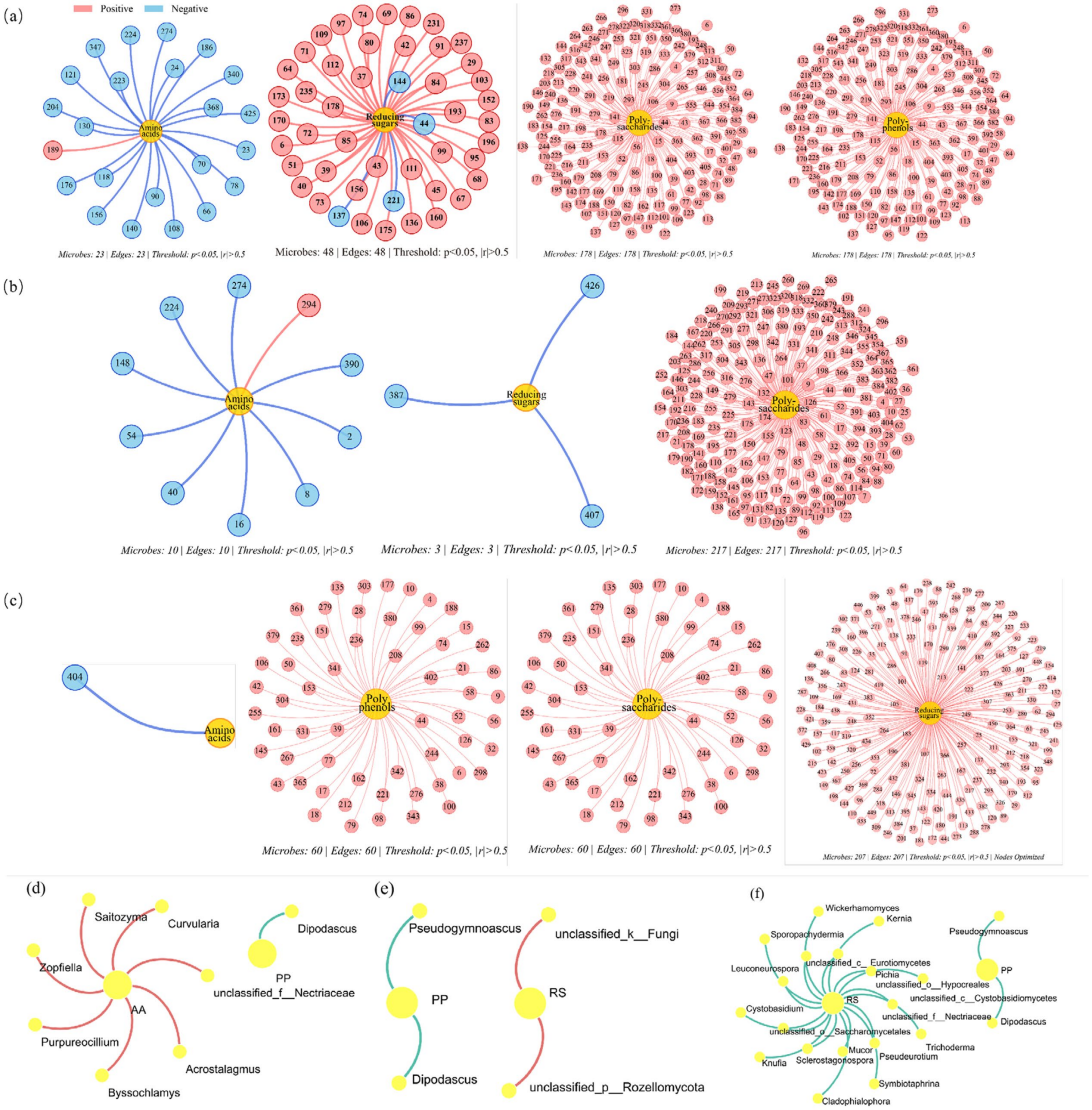
Microorganism changes associated with organic components in bacteria (**a**-CK, **b**-L1, **c**-L2) and fungi (**d**-CK, **e**-L1, **f**-L2). The bacterial names corresponding to bacterial IDs are provided in Supplementary Table S1. CK: Ordinary sludge compost without microplastics at 55 °C; L1: Ordinary sludge compost with 10% microplastics added at 55 °C; L2: High-temperature sludge compost with 10% microplastics added at 70 °C.

Microorganisms that were significantly correlated with organic components were selected for network analysis (*p* < 0.05) to further assess the co-occurrence patterns of core bacteria related to organic components in the CK, L1, and L2 groups (Zhang et al., 2022). Microorganisms at the genus level were selected to reveal the effects of MP addition and composting temperature on bacterial and fungal communities associated with organic components. The red line indicates a significant negative correlation with organic components, while the green line indicates a significant positive correlation with organic components. Each node represents a genus. The microbial community network analysis is shown in Figure 8 and Supplementary Table S1. For bacteria (Figures 8a–c), the numbers of nodes associated with organic components in the CK, L1, and L2 groups were 168, 160, and 185, respectively, while the corresponding numbers of edges were 172, 158, and 182. For fungi (Figures 8d–f), the numbers of nodes associated with organic components in the CK, L1, and L2 groups were 10, 6, and 22, respectively, with 8, 4, and 20 corresponding edges.

### 3.5 Structural equation modeling and scanning electron microscopy observation of PLA-MP degradation

**Figure 9:**
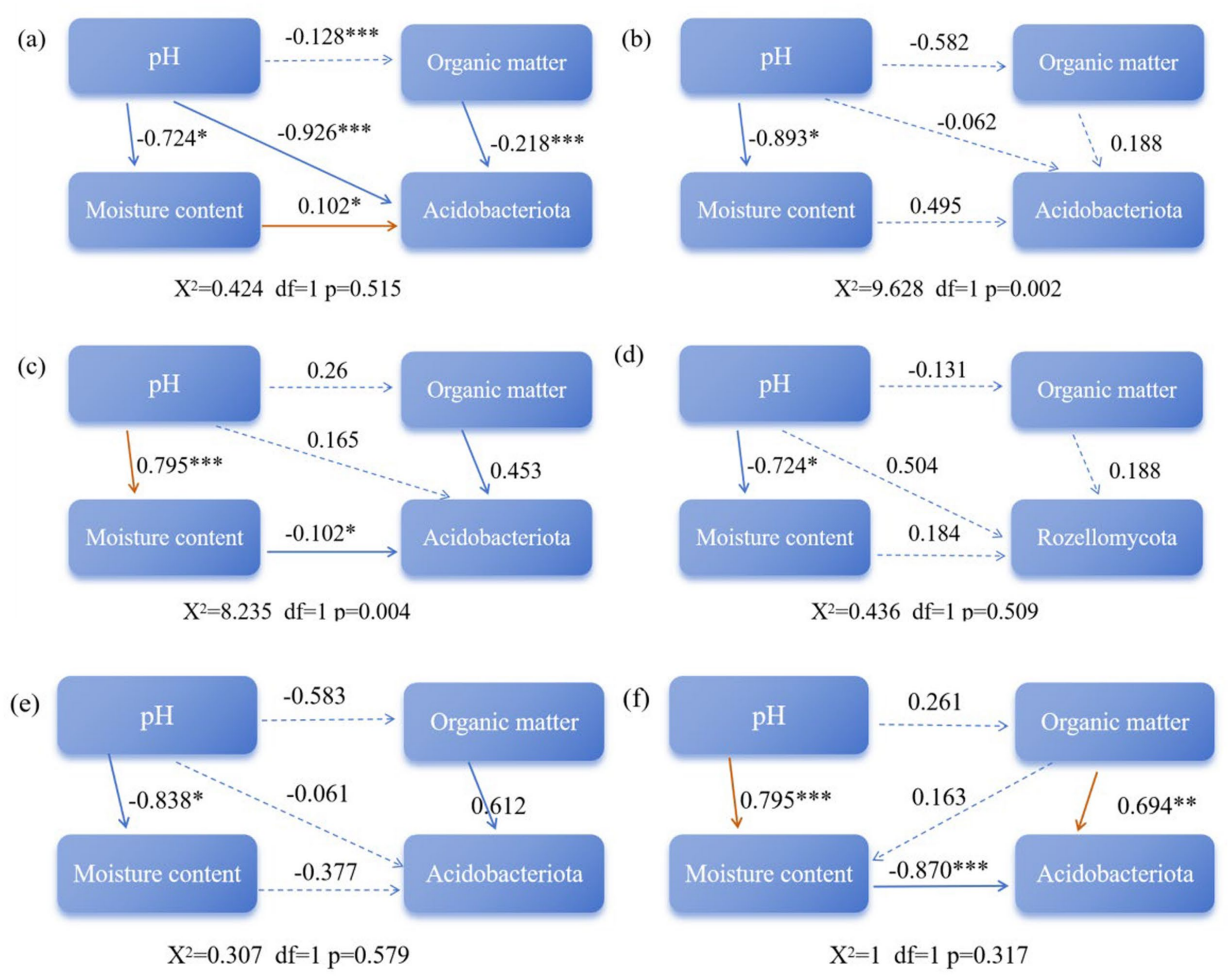
Structural equation modeling illustrating the impact of bacteria (**a**-CK, **b**-L1, **c**-L2) and fungi (**d**-CK, **e**-L1, **f**-L2) on microbial regulatory mechanisms during the composting process. CK: Ordinary sludge compost without microplastics at 55 °C; L1: Ordinary sludge compost with 10% microplastics added at 55 °C; L2: High-temperature sludge compost with 10% microplastics added at 70 °C; *p < 0.05, **p < 0.01, ***p < 0.001.

The structural equation modeling method was used to explore the relationships among multiple variables (Eisenhauer et al., 2015). As shown in Figure 9, the CK (Figure 9a), L1 (Figure 9b), and L2 treatment groups (Figure 9c) were analyzed separately. The red solid line indicates a positive correlation, the black solid line indicates a negative correlation, and the dashed line indicates no significant correlation. The corresponding numbers represent standardized path coefficients. As shown in Supplementary Figures S2a–c, after 33 d of composting, the structural equation models of the CK, L1, and L2 treatment groups were used to determine how the physicochemical factors of compost (Supplementary Figure S2) affected the relative abundance of bacteria and fungi. The model explained 84, 82, and 85% of the variation in the relative abundance of bacteria in the CK, L1, and L2 groups. pH, organic matter, and water content affected the relative abundance of Actinobacteria under different composting conditions. In the CK group, pH directly inhibited the relative abundance of Actinobacteria and also indirectly promoted it by affecting water content. In addition, organic matter also directly inhibited the relative abundance of Actinobacteria, independent of pH. In the L1 treatment group, Actinobacteria appeared to be unaffected by pH, water content, and organic matter. In the L2 treatment group, pH indirectly inhibited the relative abundance of Actinobacteria by affecting water content. The model explained 86, 82, and 70% of the variation in the relative abundance of fungi in the CK, L1, and L2 groups. As shown in Figures 9e,f, pH, organic matter, and water content affected the relative abundance of Rozellomycota under different composting conditions. Specifically, in the CK group, pH, moisture content, and organic matter had no significant effect on the relative abundance of Rozellomycota. The relative abundance of Rozellomycota in the L1 treatment group was not affected by pH, water content, or organic matter. In the L2 treatment group, pH indirectly inhibited the relative abundance of Rozellomycota by affecting water content, while organic matter directly promoted the relative abundance of Rozellomycota, independent of other factors.

Scanning electron microscopy (SEM) observations (Supplementary Figure S3) further demonstrated that high temperature was associated with enhanced MP degradation. SEM observations showed that the surface of MPs was smooth at the beginning of composting. However, after composting, obvious cracks and folds were observed on the surface of polylactic acid MPs in mesophilic compost (Chen Z. et al., 2020). After high-temperature composting, a large number of folds and large holes were observed on the surface of PLA-MPs.

## 4 Discussion

### 4.1 Temperature and MP-driven transformation of organic components

Polyphenols are a class of organic compounds widely distributed in the plant kingdom, with aromatic ring structures containing multiple hydroxyl groups. During the composting process of microplastic sludge, PP promotes aromaticity and structural stability (Zhang et al., 2015). The significantly lower PP content in the L1 and L2 groups compared to the CK group at the end of composting indicates that high temperature and the addition of MPs could inhibit PP production. This reduction might be related to enhanced microbial utilization of PP, which can promote the growth and activity of specific microorganisms. These microorganisms may possess enzymatic activity related to plastic degradation, which is associated with decreased PP content (Su et al., 2024), resulting in a stronger effect than that observed in the CK group. Therefore, polyphenols can accelerate the degradation process of microplastics by activating these microorganisms.

AAs, as easily decomposable organic nitrogen sources, are rapidly utilized by microorganisms and participate in protein synthesis and energy metabolism. The upward trend in the early and middle stages was attributed to the accelerated degradation of organic substrates by microorganisms. The significantly lower AA content in the L1 group in the later stage was possibly due to the addition of MPs, which weakened the decomposition of organic matter during composting. Under the joint participation of microplastics and high temperature, simple amino acid nitrogen was converted into complex nitrogen, which provided more favorable conditions for the accumulation of AAs and accelerated the degradation of microplastics.

RS is an important carbon source for microorganisms in compost because of its simple structure and easy absorption and utilization by microorganisms (Zhang et al., 2019). The fluctuations in RS in the L1 group indicate that the addition of MPs promoted the transformation of RS. As composting progressed, the RS produced through microbial activity was consumed as an energy source, resulting in a decrease in its content. In the L2 group, high temperature enhanced microbial activity and promoted RS accumulation.

The significant decrease in polysaccharide content at day 3 was due to their initially high levels in the early stage of composting, during which microorganisms primarily decompose polysaccharides. As composting progressed, polysaccharides were broken down into smaller molecules such as RS, resulting in a significant decrease in polysaccharide content. The overall change in polysaccharide content during composting is unstable, primarily due to the dynamic equilibrium of polysaccharide concentration as an intermediate product of organic matter decomposition. Microorganisms continuously decompose organic matter to produce polysaccharides, which are then used by microorganisms as carbon and energy sources. The slightly higher polysaccharide concentration in the L1 group compared to the CK group might be attributed to the formation of complexes between microplastics and polysaccharides, which could influence their decomposition and transformation processes. In addition, the presence of microplastics could alter the structure of microbial communities, thereby affecting the metabolic pathways of polysaccharides. The significantly higher polysaccharide content at the end of high-temperature composting was attributed to the enhanced activity of enzymes and accelerated sugar metabolism at elevated temperatures. Under these conditions, microorganisms decomposed and produced certain metabolites that could promote microplastics degradation, resulting in polysaccharide accumulation and maintenance at a high level.

Overall, the addition of MPs at high temperatures contributed to the transformation of organic components, and higher temperatures resulted in more efficient transformation. This was because adding MPs at high temperature altered the microenvironment of microbial communities and the structure of MPs, leading to the transformation of organic components.

### 4.2 Microbial community diversity and compositional shifts in response to MPs and temperature

Composting is a complex biological process that involves the transformation of organic components mediated by microorganisms. Microbial communities play a crucial role in this process, influencing the transformation of organic components. The significant reduction in the Shannon index in the L1 group indicates that MPs could inhibit the diversity of fungi and bacteria and that the inhibitory effect on fungi was stronger than that on bacteria. In contrast, the increased Shannon index in the L2 group might reflect the enhanced diversity of fungi and bacteria during high-temperature composting. The higher bacterial sequence number but lower Shannon index in the L1 group compared to the CK group suggested that the addition of MPs promoted the overall abundance of bacteria but inhibited the increase in bacterial diversity. Similarly, the lower fungal sequence number and lower Shannon index in the L1 group indicated that the addition of MPs reduced the overall abundance and diversity of fungi. Notably, the increased fungal diversity in the L2 group despite decreased abundance was due to the combined effect of MPs and temperature. These results indicate that the promoting effect of temperature on microbial diversity was stronger than the inhibitory effect of MPs on both bacterial and fungal diversity.

The NMDS results revealed that bacterial communities gathered together during the cooling and maturation periods (21 d and 33 d), indicating little difference in the bacterial community structure of different treatment groups after the high-temperature period of composting. This was likely because the high-temperature stage inhibited the growth and activity of most bacteria, and after the high-temperature period, a large number of mesophilic microorganisms grew and multiplied. The clustering of samples on days 3 and 6 reflected the differences in microbial communities caused by temperature. For fungi, unlike bacterial communities, most fungal communities were dispersed across different composting stages, and the difference was significant throughout the entire composting process. The addition of MPs might affect the community composition of microorganisms, but this process took a certain amount of time and primarily occurred in the middle and late stages of composting. As an important factor in composting experiments, temperature had a strong influence on microorganisms, making microorganisms more sensitive to temperature changes (Bialobrzewski et al., 2015).

With respect to compositional changes at the phylum level, the significantly higher Firmicutes abundance in the L2 group might be attributed to the addition of MPs and high-temperature conditions, which together promote the enrichment of this phylum (Verma et al., 2023). Actinomycetes were primarily involved in the decomposition of organic matter and were the primary bacterial community that degraded lignocellulose in aerobic compost (Yang and Li, 2024). The low abundance (0.71%) of Actinomycetes in the L1 group at day 3 was because of bacterial sensitivity to MPs. L2 delayed the response of microorganisms to MPs under high-temperature conditions. The results showed that both MP addition and high temperature inhibited the growth of Chloroflexi, whereas both factors promoted the growth of Bacteroidetes, which contributed to the degradation of organic components (Thomas et al., 2011). Moreover, the addition of MPs had little effect on other bacteria. The presence of MPs under different treatments selectively enriched microorganisms with different functions during the composting process, which inhibited the growth of other microbial groups (Zhou et al., 2022). The decreased proportion of Basidiomycota, unclassified_k__Fungi, and Rozellomycota in the L1 group indicated that the addition of microplastics inhibited the growth of these three fungi, while their significant increase in the L2 group was attributed to the promoting effect of heat on these microorganisms. The above results indicate that the composition and abundance of microbial communities undergo significant changes at different stages of composting and that the addition of MPs and high temperature could further affect the composition of microbial communities by influencing the dominant microbial populations in different composting systems.

The correlation heatmap analysis (Figure 7) showed that the addition of MPs to the compost system changed the community structure and diversity of microorganisms and indirectly affected the physicochemical factors and organic components of the compost. In addition, temperature also played a key role in microbial community structure, diversity, and bacterial richness by influencing enzyme activity. The overall pattern suggested that the addition of MPs at high temperature affected the physicochemical properties of the compost, altered the interactions between microbial communities, and was more conducive to the interactions between microbial communities and organic components, thereby accelerating the degradation process of MPs.

The decline in α diversity, accompanied by enhanced functional connectivity in the L2 group, suggested a trade-off between community complexity and specialized degradative capacity. Thermophilic conditions likely served as a strong environmental filter, selectively enriching heat-tolerant functional groups such as Firmicutes and Bacteroidetes, thereby shaping a simpler microbial community with greater metabolic redundancy. This was supported by our co-occurrence network results. The L2 group showed the largest number of nodes and edges significantly associated with organic components, with 185 nodes and 182 edges in the bacterial network and 22 nodes and 20 edges in the fungal network, despite its lower α diversity compared to the CK group. Overall, these findings indicate that reduced diversity did not impair system function; rather, it coincided with stronger organic matter degradation and higher PLA-MP biodegradation efficiency during thermophilic composting. This functional specialization also highlights the practical importance of temperature management, as appropriate thermophilic regulation might mitigate the ecological disturbance caused by MPs and improve composting performance.

### 4.3 Co-occurrence network patterns in response to MPs and temperature

Compared to the CK group, the L2 group exhibited a greater number of microbial nodes that were significantly correlated with organic components, indicating that the addition of MPs at high temperature changed the composition of key microbial communities and promoted broader microbial involvement in the turnover of organic components. A similar trend was also observed in the fungal community in the L2 treatment group. Specialized bacteria and fungi can promote the transformation of organic components (Zhao et al., 2016). The addition of MPs at high temperature increased the number of microorganisms that were significantly positively correlated with organic components and other microorganisms, thereby promoting interactions between organic components and microorganisms during composting, as well as the biodegradation of MPs. In addition, further research is needed to clarify the relationship between key microbial taxa and organic components.

### 4.4 Potential reaction mechanisms of microbial communities after PLA-MP addition

The results of structural equation modeling revealed distinct regulatory pathways among the three treatment groups. Organic matter, pH, and water content influenced the relative abundance of Actinobacteria under different composting conditions. In the CK group, pH directly inhibited the relative abundance of Actinobacteria and also indirectly promoted it by altering water content. In the L1 treatment group, Actinobacteria appeared to be unaffected by pH, water content, or organic matter, which might be due to the different sensitivity of the bacteria to PLA-MPs. In the L2 treatment group, pH indirectly inhibited the relative abundance of Actinobacteria by affecting water content. For fungi, the relative abundance of Rozellomycota in the L1 treatment group was not affected by pH, water content, or organic matter, which was due to the different sensitivity of the fungi to PLA-MPs. In the L2 treatment group, pH indirectly inhibited the relative abundance of Rozellomycota by affecting water content, while organic matter directly promoted its relative abundance without mediation by other factors.

Furthermore, SEM observations of MP surface morphology (Supplementary Figure S3) demonstrated that high temperature promoted the degradation of MPs. Some physical and chemical indicators also indicated that high-temperature composting more effectively promoted MP degradation during composting (Supplementary Figures S2a–d) (Wang N. et al., 2024). The extensive surface voids and folding observed on PLA-MPs after high-temperature composting, in contrast to the relatively milder cracks after ordinary composting (Chen Z. et al., 2020), provide direct morphological evidence supporting the higher degradation efficiency under thermophilic conditions. These results collectively demonstrate that, with the progress of composting, organic components had different effects on microbial communities under different composting conditions. Compared to the CK and mesophilic treatments, the high-temperature compost treatment promoted the activity and metabolism of microorganisms, as well as compost decomposition. By reshaping the community structure and diversity of bacteria and fungi, high-temperature composting improved the degradation efficiency of biodegradable MPs, thereby enhancing the product quality of compost and optimizing the potential environmental value of MP remediation in sludge composting systems.

## 5 Conclusion

This study investigated the core regulatory mechanisms underlying microbial synergistic degradation and organic component transformation during mesophilic (55 °C) and thermophilic (70 °C) sludge composting and clarified the coupling among biodegradable PLA-MP degradation, microbial community succession, and organic component conversion. The results showed that thermophilic composting (70 °C) significantly enhanced PLA-MP degradation, as evidenced by extensive surface voids, wrinkling, and fragmentation observed by SEM, achieving superior degradation efficiency compared to mesophilic composting (55 °C). Although 10% PLA-MP addition inhibited the α diversity of both bacterial and fungal communities, high-temperature conditions counteracted this inhibitory effect, selectively enriching thermophilic functional taxa dominated by Firmicutes (74.6–98.2%) and significantly improving the transformation dynamics of key organic components, including amino acids, reducing sugars, polysaccharides, and polyphenols. In addition, thermophilic composting enhanced microbial functional connectivity and metabolic redundancy by reshaping the microbial co-occurrence network, leading to the proliferation of polysaccharide- and lignocellulose-degrading bacteria and fungi. Structural equation modeling further revealed that high-temperature conditions optimized the compost microenvironment, including pH, organic matter, and water content, thereby regulating microbial–organic component interactions and indirectly promoting PLA-MP biodegradation. These findings provide a theoretical foundation for designing thermophilic composting strategies for the removal of biodegradable MPs in sludge treatment systems. Future research should focus on validating these findings under field-scale composting conditions and exploring the synergistic application of microbial inoculants with thermophilic composting to further enhance the removal efficiency of MPs.

## Data Availability

The raw sequencing data were submitted to the NCBI database under BioProject IDs PRJNA1449181 (bacteria) and PRJNA1449975 (fungi). The data are available at https://www.ncbi.nlm.nih.gov/bioproject/PRJNA1449181 and https://www.ncbi.nlm.nih.gov/bioproject/PRJNA1449975.
