# Supplementary material for: Biodegradable microplastics influence on organic component microbial transformation during sludge composting
Source: Front Microbiol. 2026 May 12;17:1830632. doi: 10.3389/fmicb.2026.1830632 (PMC13201416; doi:10.3389/fmicb.2026.1830632)
Supplement: Supplementary file 1 [file Data_Sheet_1.docx]

Supplementary Materials

Figure S1. Dynamic trend of temperature variation in different treatment groups during sludge composting. (CK: ordinary sludge compost without microplastics at 55 ℃; L1: ordinary sludge compost with 10% microplastics added at 55 ℃; L2: high-temperature sludge compost with 10% microplastics added at 70 ℃)

Figure S2. Changes of physicochemical indexes during sludge composting in different treatment groups (a) pH (b) OM (c) nitrate nitrogen (d) ammonium nitrogen (e) moisture content. (CK: ordinary sludge compost without microplastics at 55 ℃; L1: ordinary sludge compost with 10% microplastics added at 55 ℃; L2: high-temperature sludge compost with 10% microplastics added at 70 ℃)

Figure S3. Scanning electron microscope (SEM) image of microplastics.

Table S1. Bacterial taxa associated with organic components across different treatments and sampling times.


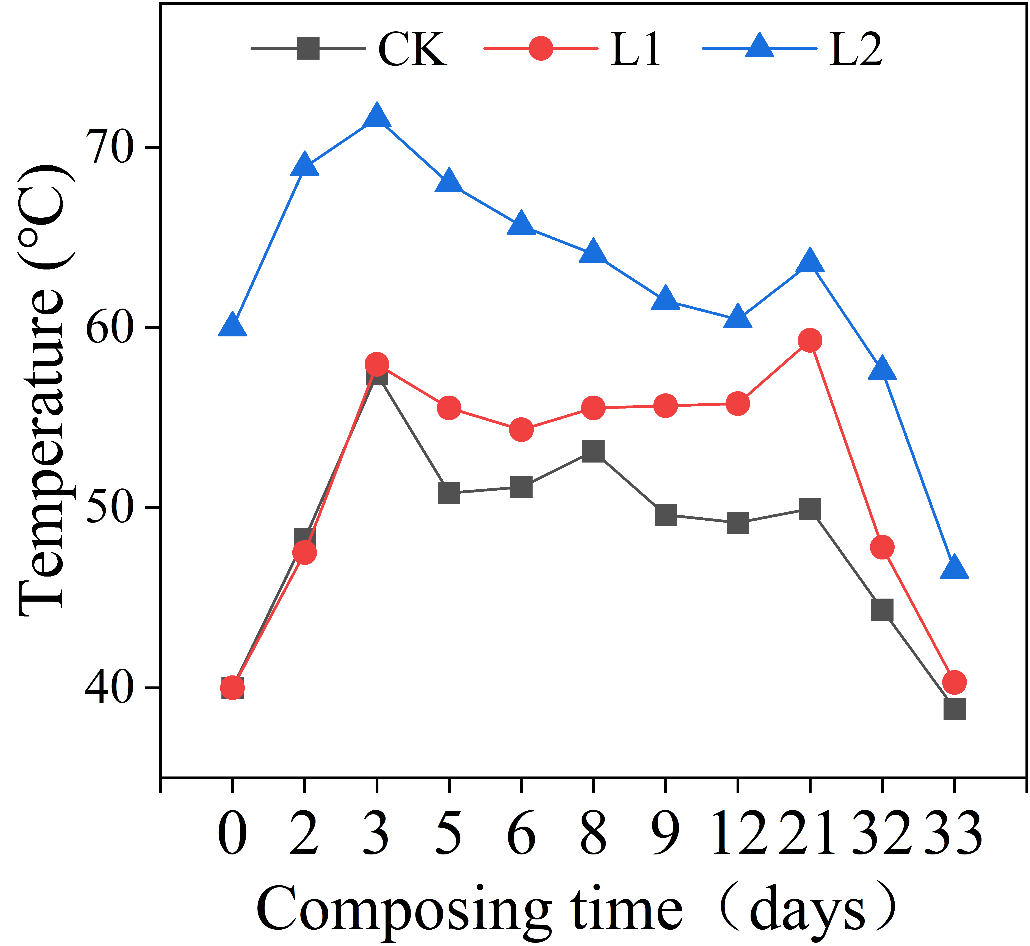


Figure.S1. Dynamic trend of temperature variation in different treatment groups during sludge composting (CK: ordinary sludge compost without microplastics at 55 ℃; L1: ordinary sludge compost with 10% microplastics added at 55 ℃; L2: high-temperature sludge compost with 10% microplastics added at 70 ℃)


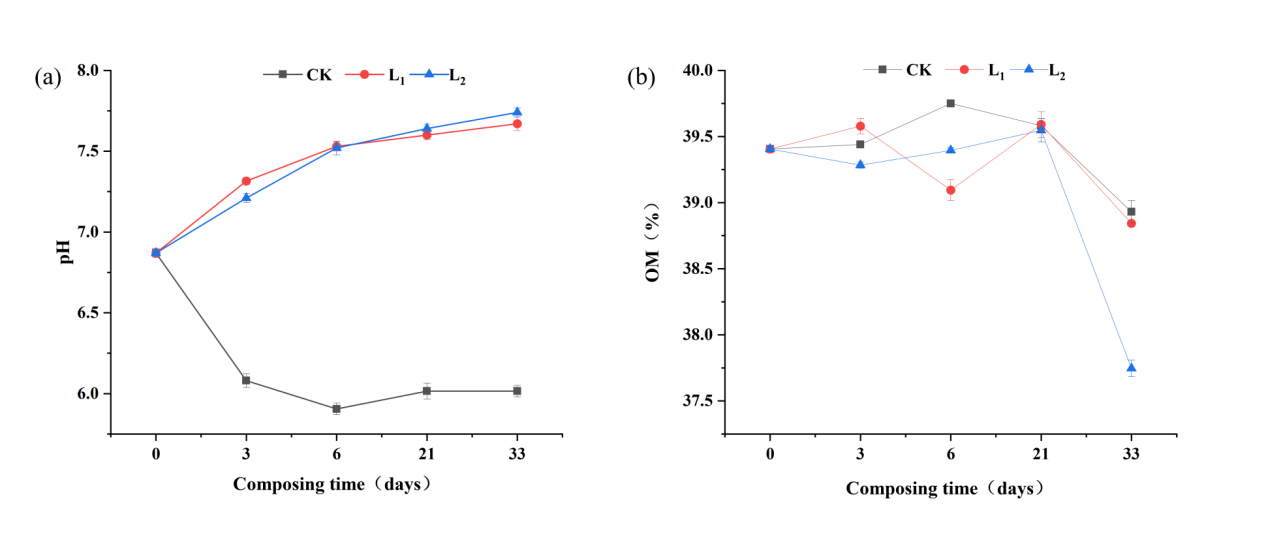


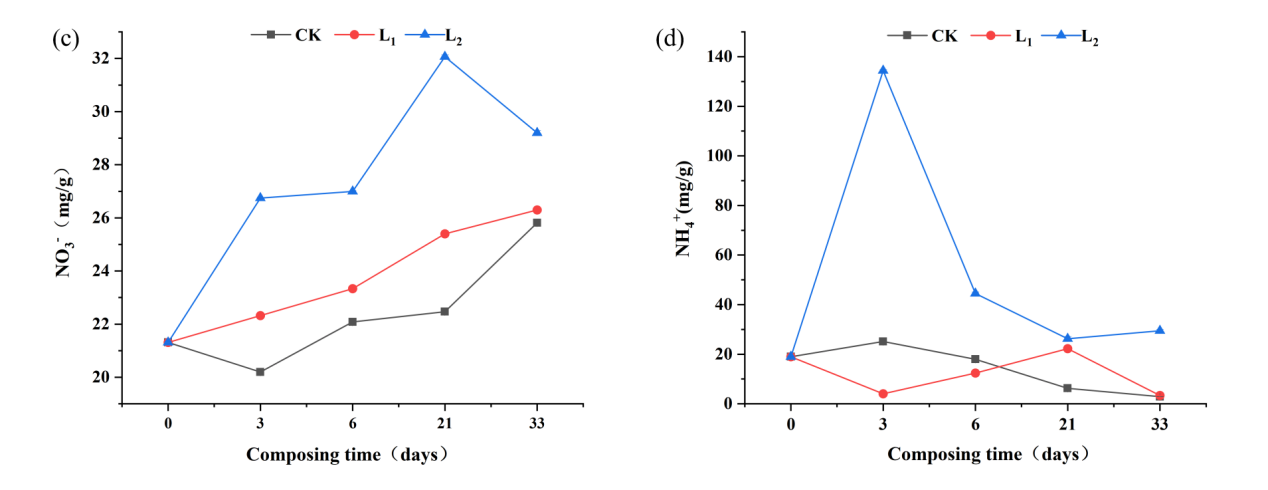


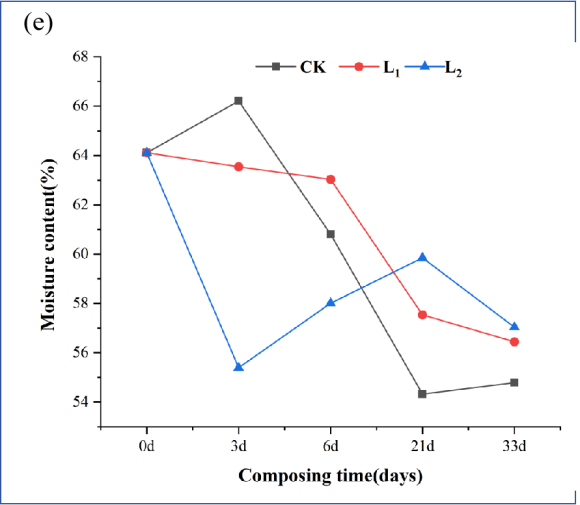


Figure.S2. Changes of physicochemical indexes during sludge composting in different treatment groups (a) pH (b) OM (c) nitrate nitrogen (d) ammonium nitrogen (e) moisture content. (CK: ordinary sludge compost without microplastics at 55 ℃; L1: ordinary sludge compost with 10% microplastics added at 55 ℃; L2: high-temperature sludge compost with 10% microplastics added at 70 ℃)


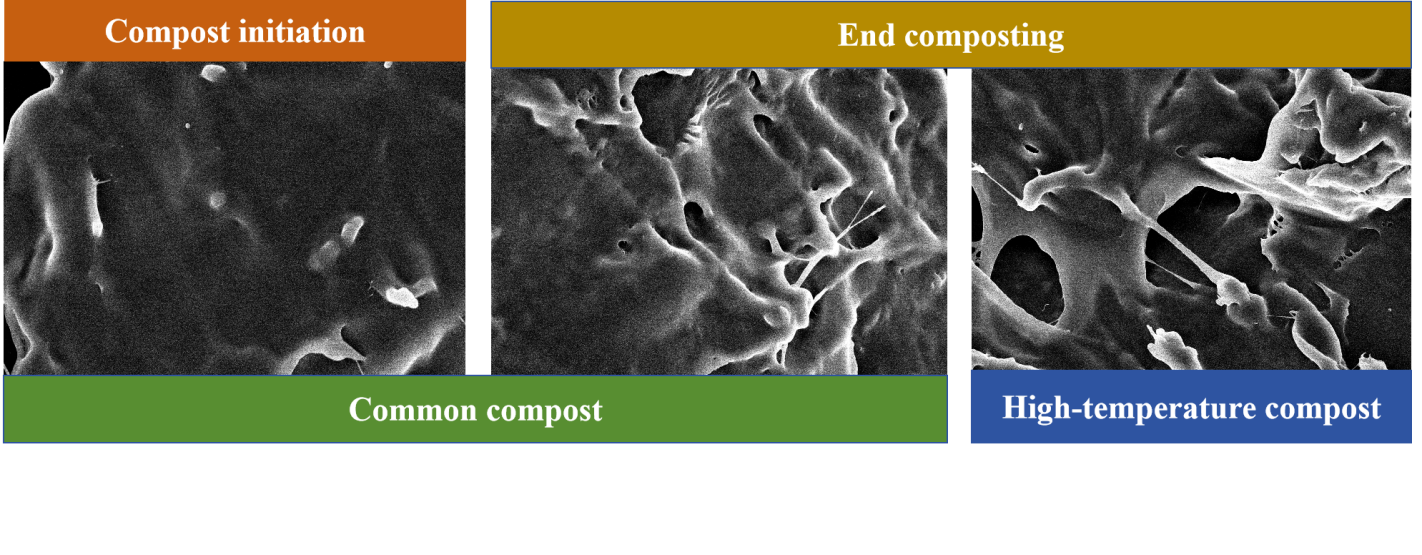


Figure.S3. Scanning electron microscope (SEM) image of microplastics

Table.S1. Bacterial taxa associated with organic components across different treatments and sampling times.

| *No.* | 0d | CK-21 | CK-3 | CK-33 | CK-6 | L1-21 | L1-3 | L1-33 | L1-6 | L2-21 | L2-3 | L2-33 | L2-6 |
| --- | --- | --- | --- | --- | --- | --- | --- | --- | --- | --- | --- | --- | --- |
| *1* |  | *Bacillus* | *Bacillus* | *Bacillus* | *Bacillus* | *Bacillus* | *Bacillus* | *Bacillus* | *Bacillus* | *Bacillus* | *Bacillus* | *Bacillus* | *Bacillus* |
| *2* | *JG30-KF-CM45* | *JG30-KF-CM45* | *JG30-KF-CM45* | *JG30-KF-CM45* | *JG30-KF-CM45* | *JG30-KF-CM45* | *JG30-KF-CM45* | *JG30-KF-CM45* | *JG30-KF-CM45* | *JG30-KF-CM45* | *JG30-KF-CM45* | *JG30-KF-CM45* | *JG30-KF-CM45* |
| *3* | *Streptomyces* | *Streptomyces* | *Streptomyces* | *Streptomyces* | *Streptomyces* | *Streptomyces* |  | *Streptomyces* | *Streptomyces* | *Streptomyces* | *Streptomyces* | *Streptomyces* | *Streptomyces* |
| *4* | *Trichococcus* |  | *Trichococcus* |  | *Trichococcus* | *Trichococcus* | *Trichococcus* |  | *Trichococcus* | *Trichococcus* | *Trichococcus* | *Trichococcus* | *Trichococcus* |
| *5* | *Sporosarcina* | *Sporosarcina* | *Sporosarcina* |  | *Sporosarcina* | *Sporosarcina* | *Sporosarcina* | *Sporosarcina* | *Sporosarcina* | *Sporosarcina* |  | *Sporosarcina* |  |
| *6* | *Enterococcus* | *Enterococcus* | *Enterococcus* | *Enterococcus* | *Enterococcus* | *Enterococcus* | *Enterococcus* | *Enterococcus* | *Enterococcus* | *Enterococcus* | *Enterococcus* | *Enterococcus* | *Enterococcus* |
| *7* | *Planococcus* | *Planococcus* | *Planococcus* |  | *Planococcus* | *Planococcus* | *Planococcus* |  | *Planococcus* | *Planococcus* | *Planococcus* | *Planococcus* | *Planococcus* |
| *8* | *Ornithinicoccus* | *Ornithinicoccus* |  | *Ornithinicoccus* | *Ornithinicoccus* | *Ornithinicoccus* |  | *Ornithinicoccus* | *Ornithinicoccus* | *Ornithinicoccus* | *Ornithinicoccus* | *Ornithinicoccus* | *Ornithinicoccus* |
| *9* | *Cellulomonadaceae* | *Cellulomonadaceae* | *Cellulomonadaceae* | *Cellulomonadaceae* | *Cellulomonadaceae* |  |  | *Cellulomonadaceae* |  | *Cellulomonadaceae* | *Cellulomonadaceae* | *Cellulomonadaceae* |  |
| *10* | *Glutamicibacter* | *Glutamicibacter* | *Glutamicibacter* | *Glutamicibacter* | *Glutamicibacter* |  |  | *Glutamicibacter* | *Glutamicibacter* |  | *Glutamicibacter* |  |  |
| *11* |  | *Rhodothermaceae* |  | *Rhodothermaceae* |  | *Rhodothermaceae* |  | *Rhodothermaceae* |  | *Rhodothermaceae* |  | *Rhodothermaceae* |  |
| *12* |  | *Saccharomonospora* |  | *Saccharomonospora* | *Saccharomonospora* | *Saccharomonospora* |  | *Saccharomonospora* | *Saccharomonospora* | *Saccharomonospora* |  | *Saccharomonospora* | *Saccharomonospora* |
| *13* |  | *Actinomadura* |  | *Actinomadura* | *Actinomadura* | *Actinomadura* |  | *Actinomadura* | *Actinomadura* | *Actinomadura* |  | *Actinomadura* | *Actinomadura* |
| *14* |  | *Ureibacillus* | *Ureibacillus* | *Ureibacillus* | *Ureibacillus* | *Ureibacillus* | *Ureibacillus* | *Ureibacillus* | *Ureibacillus* | *Ureibacillus* | *Ureibacillus* | *Ureibacillus* | *Ureibacillus* |
| *15* | *Proteiniclasticum* |  | *Proteiniclasticum* |  |  | *Proteiniclasticum* |  | *Proteiniclasticum* |  |  | *Proteiniclasticum* |  | *Proteiniclasticum* |
| *16* | *Georgenia* | *Georgenia* | *Georgenia* | *Georgenia* | *Georgenia* | *Georgenia* |  | *Georgenia* |  | *Georgenia* | *Georgenia* | *Georgenia* | *Georgenia* |
| *17* | *Carnobacterium* |  | *Carnobacterium* |  | *Carnobacterium* |  | *Carnobacterium* |  |  |  | *Carnobacterium* |  |  |
| *18* | *Jeotgalibaca* |  | *Jeotgalibaca* |  |  |  | *Jeotgalibaca* |  | *Jeotgalibaca* |  | *Jeotgalibaca* |  | *Jeotgalibaca* |
| *19* | *Paenibacillus* | *Paenibacillus* | *Paenibacillus* | *Paenibacillus* | *Paenibacillus* | *Paenibacillus* | *Paenibacillus* | *Paenibacillus* | *Paenibacillus* | *Paenibacillus* | *Paenibacillus* | *Paenibacillus* | *Paenibacillus* |
| *20* | *Romboutsia* | *Romboutsia* | *Romboutsia* | *Romboutsia* | *Romboutsia* | *Romboutsia* | *Romboutsia* | *Romboutsia* | *Romboutsia* | *Romboutsia* | *Romboutsia* | *Romboutsia* | *Romboutsia* |
| *21* | *Brachybacterium* | *Brachybacterium* | *Brachybacterium* |  | *Brachybacterium* |  | *Brachybacterium* |  | *Brachybacterium* | *Brachybacterium* | *Brachybacterium* | *Brachybacterium* |  |
| *22* |  | *Virgibacillus* | *Virgibacillus* | *Virgibacillus* | *Virgibacillus* | *Virgibacillus* | *Virgibacillus* | *Virgibacillus* | *Virgibacillus* | *Virgibacillus* |  |  |  |
| *23* |  | *S0134_terrestrial_group* |  | *S0134_terrestrial_group* |  | *S0134_terrestrial_group* |  | *S0134_terrestrial_group* | *S0134_terrestrial_group* | *S0134_terrestrial_group* |  | *S0134_terrestrial_group* |  |
| *24* |  |  |  | *Geobacillus* |  | *Geobacillus* |  |  |  | *Geobacillus* | *Geobacillus* |  | *Geobacillus* |
| *25* | *Microtrichales2* | *Microtrichales2* | *Microtrichales2* | *Microtrichales2* | *Microtrichales2* | *Microtrichales2* | *Microtrichales2* | *Microtrichales2* | *Microtrichales2* | *Microtrichales2* | *Microtrichales2* | *Microtrichales2* | *Microtrichales2* |
| *26* |  | *Bacillaceae2* | *Bacillaceae2* | *Bacillaceae2* | *Bacillaceae2* | *Bacillaceae2* | *Bacillaceae2* | *Bacillaceae2* | *Bacillaceae2* | *Bacillaceae2* | *Bacillaceae2* | *Bacillaceae2* | *Bacillaceae2* |
| *27* | *Saccharimonadales2* | *Saccharimonadales2* | *Saccharimonadales2* | *Saccharimonadales2* | *Saccharimonadales2* | *Saccharimonadales2* | *Saccharimonadales2* | *Saccharimonadales2* | *Saccharimonadales2* | *Saccharimonadales2* | *Saccharimonadales2* | *Saccharimonadales2* | *Saccharimonadales2* |
| *28* | *Yersinia* |  |  |  |  |  |  |  |  |  |  |  |  |
| *29* | *Micropruina* | *Micropruina* | *Micropruina* | *Micropruina* | *Micropruina* | *Micropruina* | *Micropruina* | *Micropruina* | *Micropruina* |  | *Micropruina* | *Micropruina* | *Micropruina* |
| *30* | *Oceanobacillus* | *Oceanobacillus* | *Oceanobacillus* | *Oceanobacillus* | *Oceanobacillus* | *Oceanobacillus* | *Oceanobacillus* | *Oceanobacillus* | *Oceanobacillus* | *Oceanobacillus* | *Oceanobacillus* | *Oceanobacillus* | *Oceanobacillus* |
| *31* |  | *Kroppenstedtia* |  | *Kroppenstedtia* | *Kroppenstedtia* | *Kroppenstedtia* |  | *Kroppenstedtia* | *Kroppenstedtia* | *Kroppenstedtia* |  | *Kroppenstedtia* | *Kroppenstedtia* |
| *32* | *Lactobacillus* |  | *Lactobacillus* |  | *Lactobacillus* |  |  | *Lactobacillus* | *Lactobacillus* | *Lactobacillus* | *Lactobacillus* | *Lactobacillus* |  |
| *33* |  | *Aeribacillus* | *Aeribacillus* | *Aeribacillus* | *Aeribacillus* | *Aeribacillus* |  | *Aeribacillus* | *Aeribacillus* | *Aeribacillus* | *Aeribacillus* | *Aeribacillus* | *Aeribacillus* |
| *34* |  | *Pseudactinotalea* | *Pseudactinotalea* | *Pseudactinotalea* | *Pseudactinotalea* | *Pseudactinotalea* |  | *Pseudactinotalea* | *Pseudactinotalea* | *Pseudactinotalea* |  | *Pseudactinotalea* |  |
| *35* |  | *Psychrobacillus* | *Psychrobacillus* |  | *Psychrobacillus* |  | *Psychrobacillus* |  |  |  | *Psychrobacillus* |  |  |
| *36* | *Hyphomicrobium* | *Hyphomicrobium* | *Hyphomicrobium* | *Hyphomicrobium* | *Hyphomicrobium* | *Hyphomicrobium* | *Hyphomicrobium* | *Hyphomicrobium* | *Hyphomicrobium* | *Hyphomicrobium* | *Hyphomicrobium* | *Hyphomicrobium* | *Hyphomicrobium* |
| *37* | *67-14* | *67-14* | *67-14* | *67-14* | *67-14* | *67-14* | *67-14* | *67-14* | *67-14* | *67-14* | *67-14* | *67-14* | *67-14* |
| *38* | *Desemzia* |  | *Desemzia* |  | *Desemzia* | *Desemzia* | *Desemzia* |  | *Desemzia* |  | *Desemzia* |  |  |
| *39* | *Macellibacteroides* |  |  |  |  |  |  |  |  |  | *Macellibacteroides* |  | *Macellibacteroides* |
| *40* |  | *Chelativorans* |  | *Chelativorans* | *Chelativorans* | *Chelativorans* |  | *Chelativorans* |  | *Chelativorans* | *Chelativorans* | *Chelativorans* |  |
| *41* | *Clostridium_sensu_stricto_1* | *Clostridium_sensu_stricto_1* | *Clostridium_sensu_stricto_1* | *Clostridium_sensu_stricto_1* | *Clostridium_sensu_stricto_1* | *Clostridium_sensu_stricto_1* | *Clostridium_sensu_stricto_1* | *Clostridium_sensu_stricto_1* | *Clostridium_sensu_stricto_1* | *Clostridium_sensu_stricto_1* | *Clostridium_sensu_stricto_1* | *Clostridium_sensu_stricto_1* | *Clostridium_sensu_stricto_1* |
| *42* | *Acinetobacter* |  |  |  |  |  |  |  |  | *Acinetobacter* | *Acinetobacter* | *Acinetobacter* | *Acinetobacter* |
| *43* | *Hafnia-Obesumbacterium* |  |  |  |  |  |  |  |  | *Hafnia-Obesumbacterium* | *Hafnia-Obesumbacterium* |  | *Hafnia-Obesumbacterium* |
| *44* | *Tissierella* | *Tissierella* |  | *Tissierella* |  |  |  | *Tissierella* | *Tissierella* | *Tissierella* |  | *Tissierella* |  |
| *45* |  | *Citricoccus* | *Citricoccus* |  | *Citricoccus* |  |  |  | *Citricoccus* |  |  |  |  |
| *46* |  | *Bacilli* | *Bacilli* | *Bacilli* | *Bacilli* | *Bacilli* | *Bacilli* | *Bacilli* | *Bacilli* |  |  |  |  |
| *47* | *Propioniciclava* | *Propioniciclava* | *Propioniciclava* | *Propioniciclava* | *Propioniciclava* | *Propioniciclava* | *Propioniciclava* | *Propioniciclava* | *Propioniciclava* | *Propioniciclava* | *Propioniciclava* | *Propioniciclava* | *Propioniciclava* |
| *48* | *PeM15* | *PeM15* | *PeM15* | *PeM15* | *PeM15* |  | *PeM15* | *PeM15* | *PeM15* | *PeM15* | *PeM15* | *PeM15* | *PeM15* |
| *49* | *Mycobacterium* | *Mycobacterium* | *Mycobacterium* | *Mycobacterium* | *Mycobacterium* | *Mycobacterium* | *Mycobacterium* | *Mycobacterium* | *Mycobacterium* | *Mycobacterium* | *Mycobacterium* | *Mycobacterium* | *Mycobacterium* |
| *50* | *Corynebacterium* | *Corynebacterium* | *Corynebacterium* |  | *Corynebacterium* |  |  | *Corynebacterium* |  |  | *Corynebacterium* |  |  |
| *51* | *Micromonospora* | *Micromonospora* | *Micromonospora* | *Micromonospora* | *Micromonospora* | *Micromonospora* | *Micromonospora* | *Micromonospora* | *Micromonospora* | *Micromonospora* | *Micromonospora* | *Micromonospora* | *Micromonospora* |
| *52* | *Proteiniphilum* |  |  |  |  |  |  |  |  |  |  |  |  |
| *53* | *KD4-96* | *KD4-96* | *KD4-96* | *KD4-96* | *KD4-96* | *KD4-96* | *KD4-96* | *KD4-96* | *KD4-96* | *KD4-96* | *KD4-96* | *KD4-96* | *KD4-96* |
| *54* |  | *Methylococcaceae* |  | *Methylococcaceae* |  | *Methylococcaceae* |  | *Methylococcaceae* |  | *Methylococcaceae* |  | *Methylococcaceae* | *Methylococcaceae* |
| *55* |  | *Vulgatibacter* |  | *Vulgatibacter* |  | *Vulgatibacter* |  | *Vulgatibacter* |  | *Vulgatibacter* |  | *Vulgatibacter* | *Vulgatibacter* |
| *56* | *Citrobacter* |  |  |  |  |  |  |  |  | *Citrobacter* | *Citrobacter* |  | *Citrobacter* |
| *57* | *Ralstonia* |  | *Ralstonia* |  | *Ralstonia* | *Ralstonia* | *Ralstonia* | *Ralstonia* |  | *Ralstonia* | *Ralstonia* | *Ralstonia* | *Ralstonia* |
| *58* | *Rhodococcus* | *Rhodococcus* | *Rhodococcus* | *Rhodococcus* | *Rhodococcus* | *Rhodococcus* | *Rhodococcus* | *Rhodococcus* | *Rhodococcus* | *Rhodococcus* | *Rhodococcus* | *Rhodococcus* | *Rhodococcus* |
| *59* |  | *Thermobacillus* |  | *Thermobacillus* | *Thermobacillus* | *Thermobacillus* |  | *Thermobacillus* | *Thermobacillus* | *Thermobacillus* |  | *Thermobacillus* | *Thermobacillus* |
| *60* | *Hyphomicrobiaceae1* | *Hyphomicrobiaceae1* | *Hyphomicrobiaceae1* | *Hyphomicrobiaceae1* | *Hyphomicrobiaceae1* | *Hyphomicrobiaceae1* | *Hyphomicrobiaceae1* | *Hyphomicrobiaceae1* | *Hyphomicrobiaceae1* | *Hyphomicrobiaceae1* | *Hyphomicrobiaceae1* | *Hyphomicrobiaceae1* | *Hyphomicrobiaceae1* |
| *61* | *Ornithinibacter* |  | *Ornithinibacter* |  | *Ornithinibacter* | *Ornithinibacter* | *Ornithinibacter* | *Ornithinibacter* | *Ornithinibacter* | *Ornithinibacter* | *Ornithinibacter* | *Ornithinibacter* | *Ornithinibacter* |
| *62* | *Conexibacter* | *Conexibacter* | *Conexibacter* | *Conexibacter* | *Conexibacter* | *Conexibacter* | *Conexibacter* | *Conexibacter* | *Conexibacter* | *Conexibacter* | *Conexibacter* | *Conexibacter* | *Conexibacter* |
| *63* |  | *Novibacillus* |  | *Novibacillus* | *Novibacillus* | *Novibacillus* |  | *Novibacillus* | *Novibacillus* | *Novibacillus* |  | *Novibacillus* | *Novibacillus* |
| *64* | *IMCC26207* | *IMCC26207* | *IMCC26207* | *IMCC26207* | *IMCC26207* | *IMCC26207* | *IMCC26207* | *IMCC26207* | *IMCC26207* | *IMCC26207* | *IMCC26207* | *IMCC26207* | *IMCC26207* |
| *65* |  | *Brevibacillus* |  | *Brevibacillus* | *Brevibacillus* | *Brevibacillus* |  | *Brevibacillus* | *Brevibacillus* | *Brevibacillus* |  | *Brevibacillus* | *Brevibacillus* |
| *66* |  |  |  | *Ammoniphilus* |  | *Ammoniphilus* |  | *Ammoniphilus* | *Ammoniphilus* | *Ammoniphilus* |  | *Ammoniphilus* |  |
| *67* | *Paraclostridium* | *Paraclostridium* | *Paraclostridium* | *Paraclostridium* | *Paraclostridium* | *Paraclostridium* | *Paraclostridium* | *Paraclostridium* | *Paraclostridium* | *Paraclostridium* | *Paraclostridium* | *Paraclostridium* | *Paraclostridium* |
| *68* | *Exiguobacterium* |  | *Exiguobacterium* |  | *Exiguobacterium* | *Exiguobacterium* | *Exiguobacterium* | *Exiguobacterium* | *Exiguobacterium* | *Exiguobacterium* | *Exiguobacterium* | *Exiguobacterium* | *Exiguobacterium* |
| *69* | *Blastocatellaceae1* |  | *Blastocatellaceae1* | *Blastocatellaceae1* |  | *Blastocatellaceae1* | *Blastocatellaceae1* | *Blastocatellaceae1* | *Blastocatellaceae1* | *Blastocatellaceae1* | *Blastocatellaceae1* | *Blastocatellaceae1* | *Blastocatellaceae1* |
| *70* | *Microbacterium* | *Microbacterium* | *Microbacterium* | *Microbacterium* | *Microbacterium* | *Microbacterium* | *Microbacterium* | *Microbacterium* |  | *Microbacterium* | *Microbacterium* | *Microbacterium* |  |
| *71* | *Caldilineaceae* | *Caldilineaceae* | *Caldilineaceae* | *Caldilineaceae* | *Caldilineaceae* | *Caldilineaceae* | *Caldilineaceae* | *Caldilineaceae* |  | *Caldilineaceae* | *Caldilineaceae* | *Caldilineaceae* | *Caldilineaceae* |
| *72* | *Propionicimonas* | *Propionicimonas* | *Propionicimonas* |  | *Propionicimonas* | *Propionicimonas* | *Propionicimonas* | *Propionicimonas* | *Propionicimonas* | *Propionicimonas* | *Propionicimonas* | *Propionicimonas* | *Propionicimonas* |
| *73* |  | *Paenibacillaceae* |  | *Paenibacillaceae* |  | *Paenibacillaceae* |  | *Paenibacillaceae* |  | *Paenibacillaceae* |  | *Paenibacillaceae* |  |
| *74* | *Clostridium_sensu_stricto_13* |  | *Clostridium_sensu_stricto_13* |  | *Clostridium_sensu_stricto_13* |  | *Clostridium_sensu_stricto_13* |  | *Clostridium_sensu_stricto_13* | *Clostridium_sensu_stricto_13* | *Clostridium_sensu_stricto_13* | *Clostridium_sensu_stricto_13* |  |
| *75* |  | *Thermomonosporaceae* |  | *Thermomonosporaceae* |  | *Thermomonosporaceae* |  | *Thermomonosporaceae* | *Thermomonosporaceae* | *Thermomonosporaceae* |  |  |  |
| *76* | *Turicibacter* | *Turicibacter* | *Turicibacter* | *Turicibacter* | *Turicibacter* | *Turicibacter* | *Turicibacter* | *Turicibacter* | *Turicibacter* | *Turicibacter* | *Turicibacter* | *Turicibacter* | *Turicibacter* |
| *77* | *Lactobacillales* |  |  |  |  |  |  |  |  |  |  |  |  |
| *78* |  | *Microvirga* |  | *Microvirga* |  | *Microvirga* |  | *Microvirga* |  | *Microvirga* |  | *Microvirga* |  |
| *79* | *Leuconostoc* |  | *Leuconostoc* |  |  |  |  |  |  |  | *Leuconostoc* |  |  |
| *80* | *Nocardioides* |  | *Nocardioides* | *Nocardioides* | *Nocardioides* | *Nocardioides* | *Nocardioides* | *Nocardioides* | *Nocardioides* | *Nocardioides* | *Nocardioides* | *Nocardioides* | *Nocardioides* |
| *81* |  | *Bacillaceae1* |  | *Bacillaceae1* | *Bacillaceae1* | *Bacillaceae1* |  | *Bacillaceae1* | *Bacillaceae1* | *Bacillaceae1* | *Bacillaceae1* | *Bacillaceae1* | *Bacillaceae1* |
| *82* | *Propionicicella* |  |  |  |  | *Propionicicella* |  | *Propionicicella* | *Propionicicella* |  | *Propionicicella* | *Propionicicella* | *Propionicicella* |
| *83* | *Brooklawnia* | *Brooklawnia* | *Brooklawnia* | *Brooklawnia* | *Brooklawnia* | *Brooklawnia* | *Brooklawnia* | *Brooklawnia* | *Brooklawnia* | *Brooklawnia* | *Brooklawnia* | *Brooklawnia* | *Brooklawnia* |
| *84* | *Iamia* |  | *Iamia* |  |  |  | *Iamia* | *Iamia* | *Iamia* | *Iamia* | *Iamia* | *Iamia* | *Iamia* |
| *85* | *Gordonia* | *Gordonia* | *Gordonia* | *Gordonia* | *Gordonia* | *Gordonia* | *Gordonia* | *Gordonia* | *Gordonia* | *Gordonia* | *Gordonia* | *Gordonia* | *Gordonia* |
| *86* | *Proteocatella* |  |  |  |  |  |  |  |  |  | *Proteocatella* |  |  |
| *87* |  | *Bhargavaea* |  | *Bhargavaea* | *Bhargavaea* | *Bhargavaea* | *Bhargavaea* | *Bhargavaea* | *Bhargavaea* | *Bhargavaea* |  | *Bhargavaea* |  |
| *88* | *1-20* | *1-20* | *1-20* |  | *1-20* | *1-20* | *1-20* | *1-20* | *1-20* |  | *1-20* | *1-20* | *1-20* |
| *89* | *Candidatus_Microthrix* |  | *Candidatus_Microthrix* |  |  |  | *Candidatus_Microthrix* |  | *Candidatus_Microthrix* | *Candidatus_Microthrix* | *Candidatus_Microthrix* | *Candidatus_Microthrix* | *Candidatus_Microthrix* |
| *90* |  | *Pusillimonas* |  | *Pusillimonas* |  |  |  | *Pusillimonas* |  |  |  | *Pusillimonas* |  |
| *91* | *Legionella* |  | *Legionella* |  |  | *Legionella* | *Legionella* |  | *Legionella* | *Legionella* | *Legionella* | *Legionella* | *Legionella* |
| *92* | *Propionibacteriaceae1* |  | *Propionibacteriaceae1* | *Propionibacteriaceae1* | *Propionibacteriaceae1* |  | *Propionibacteriaceae1* | *Propionibacteriaceae1* | *Propionibacteriaceae1* |  | *Propionibacteriaceae1* | *Propionibacteriaceae1* | *Propionibacteriaceae1* |
| *93* | *Marmoricola* | *Marmoricola* | *Marmoricola* | *Marmoricola* | *Marmoricola* | *Marmoricola* | *Marmoricola* | *Marmoricola* |  |  | *Marmoricola* | *Marmoricola* |  |
| *94* | *Rhizobiales_Incertae_Sedis* |  | *Rhizobiales_Incertae_Sedis* |  | *Rhizobiales_Incertae_Sedis* |  | *Rhizobiales_Incertae_Sedis* |  | *Rhizobiales_Incertae_Sedis* | *Rhizobiales_Incertae_Sedis* | *Rhizobiales_Incertae_Sedis* | *Rhizobiales_Incertae_Sedis* | *Rhizobiales_Incertae_Sedis* |
| *95* | *Gaiella* | *Gaiella* | *Gaiella* | *Gaiella* |  |  | *Gaiella* | *Gaiella* | *Gaiella* | *Gaiella* | *Gaiella* | *Gaiella* | *Gaiella* |
| *96* | *Gaiellales* | *Gaiellales* | *Gaiellales* | *Gaiellales* | *Gaiellales* |  | *Gaiellales* | *Gaiellales* | *Gaiellales* | *Gaiellales* | *Gaiellales* | *Gaiellales* | *Gaiellales* |
| *97* | *Desulfosporosinus* | *Desulfosporosinus* |  | *Desulfosporosinus* |  | *Desulfosporosinus* |  |  |  | *Desulfosporosinus* | *Desulfosporosinus* | *Desulfosporosinus* | *Desulfosporosinus* |
| *98* | *Anaerocolumna* | *Anaerocolumna* |  |  |  |  |  |  |  |  |  | *Anaerocolumna* | *Anaerocolumna* |
| *99* | *Aerococcaceae* |  |  |  |  |  |  |  |  |  |  |  |  |
| *100* | *OPB41* |  |  |  |  |  |  |  | *OPB41* |  | *OPB41* |  |  |
| *101* | *Actinomycetaceae1* |  | *Actinomycetaceae1* | *Actinomycetaceae1* | *Actinomycetaceae1* | *Actinomycetaceae1* | *Actinomycetaceae1* | *Actinomycetaceae1* | *Actinomycetaceae1* |  | *Actinomycetaceae1* | *Actinomycetaceae1* |  |
| *102* | *Anaerolineaceae1* |  | *Anaerolineaceae1* | *Anaerolineaceae1* |  |  | *Anaerolineaceae1* | *Anaerolineaceae1* | *Anaerolineaceae1* | *Anaerolineaceae1* | *Anaerolineaceae1* |  | *Anaerolineaceae1* |
| *103* |  | *Bacillales* |  | *Bacillales* |  | *Bacillales* |  | *Bacillales* | *Bacillales* | *Bacillales* |  | *Bacillales* |  |
| *104* |  |  |  |  |  |  | *Aquabacterium* | *Aquabacterium* |  |  | *Aquabacterium* | *Aquabacterium* | *Aquabacterium* |
| *105* | *IMCC26256* | *IMCC26256* | *IMCC26256* |  |  | *IMCC26256* | *IMCC26256* | *IMCC26256* | *IMCC26256* |  | *IMCC26256* | *IMCC26256* | *IMCC26256* |
| *106* | *Acetoanaerobium* |  |  |  |  |  |  |  |  |  |  |  |  |
| *107* | *Bradyrhizobium* |  | *Bradyrhizobium* |  | *Bradyrhizobium* |  | *Bradyrhizobium* | *Bradyrhizobium* | *Bradyrhizobium* | *Bradyrhizobium* | *Bradyrhizobium* | *Bradyrhizobium* | *Bradyrhizobium* |
| *108* |  |  |  | *Sphingomonadaceae2* |  |  |  | *Sphingomonadaceae2* |  |  | *Sphingomonadaceae2* | *Sphingomonadaceae2* |  |
| *109* | *Rhodobacter* |  | *Rhodobacter* |  |  | *Rhodobacter* | *Rhodobacter* |  | *Rhodobacter* |  | *Rhodobacter* | *Rhodobacter* | *Rhodobacter* |
| *110* | *SC-I-84* |  |  |  |  |  |  |  |  |  | *SC-I-84* |  | *SC-I-84* |
| *111* |  | *Pediococcus* | *Pediococcus* | *Pediococcus* | *Pediococcus* | *Pediococcus* | *Pediococcus* | *Pediococcus* | *Pediococcus* | *Pediococcus* |  | *Pediococcus* | *Pediococcus* |
| *112* | *Tessaracoccus* |  | *Tessaracoccus* |  |  |  | *Tessaracoccus* |  | *Tessaracoccus* |  | *Tessaracoccus* |  | *Tessaracoccus* |
| *113* | *Defluviimonas* | *Defluviimonas* | *Defluviimonas* |  |  |  | *Defluviimonas* |  | *Defluviimonas* |  | *Defluviimonas* | *Defluviimonas* | *Defluviimonas* |
| *114* | *KIST-JJY010* |  | *KIST-JJY010* |  | *KIST-JJY010* | *KIST-JJY010* | *KIST-JJY010* | *KIST-JJY010* | *KIST-JJY010* | *KIST-JJY010* | *KIST-JJY010* |  | *KIST-JJY010* |
| *115* | *A4b* |  | *A4b* |  |  |  | *A4b* |  |  |  | *A4b* | *A4b* | *A4b* |
| *116* |  | *Clostridium_sensu_stricto_7* | *Clostridium_sensu_stricto_7* | *Clostridium_sensu_stricto_7* |  | *Clostridium_sensu_stricto_7* | *Clostridium_sensu_stricto_7* | *Clostridium_sensu_stricto_7* | *Clostridium_sensu_stricto_7* | *Clostridium_sensu_stricto_7* |  | *Clostridium_sensu_stricto_7* | *Clostridium_sensu_stricto_7* |
| *117* | *Saprospiraceae* |  |  |  |  |  |  |  |  | *Saprospiraceae* | *Saprospiraceae* |  | *Saprospiraceae* |
| *118* |  | *DSSF69* |  | *DSSF69* |  | *DSSF69* |  | *DSSF69* |  |  |  | *DSSF69* |  |
| *119* | *Ilumatobacteraceae2* |  | *Ilumatobacteraceae2* |  |  | *Ilumatobacteraceae2* | *Ilumatobacteraceae2* |  |  | *Ilumatobacteraceae2* | *Ilumatobacteraceae2* |  | *Ilumatobacteraceae2* |
| *120* | *Steroidobacteraceae* |  |  |  |  |  | *Steroidobacteraceae* |  | *Steroidobacteraceae* | *Steroidobacteraceae* | *Steroidobacteraceae* |  |  |
| *121* |  |  |  | *Thermobifida* |  | *Thermobifida* |  | *Thermobifida* | *Thermobifida* | *Thermobifida* |  | *Thermobifida* | *Thermobifida* |
| *122* | *AKYG1722* | *AKYG1722* | *AKYG1722* |  | *AKYG1722* |  | *AKYG1722* |  | *AKYG1722* | *AKYG1722* | *AKYG1722* |  |  |
| *123* | *Leucobacter* | *Leucobacter* | *Leucobacter* | *Leucobacter* | *Leucobacter* |  | *Leucobacter* | *Leucobacter* | *Leucobacter* |  | *Leucobacter* | *Leucobacter* | *Leucobacter* |
| *124* | *Methyloceanibacter* | *Methyloceanibacter* | *Methyloceanibacter* | *Methyloceanibacter* | *Methyloceanibacter* | *Methyloceanibacter* | *Methyloceanibacter* | *Methyloceanibacter* | *Methyloceanibacter* |  | *Methyloceanibacter* | *Methyloceanibacter* | *Methyloceanibacter* |
| *125* | *Ottowia* |  |  |  |  |  |  |  |  |  | *Ottowia* |  | *Ottowia* |
| *126* | *Dietzia* | *Dietzia* | *Dietzia* | *Dietzia* | *Dietzia* |  |  | *Dietzia* |  |  | *Dietzia* |  | *Dietzia* |
| *127* | *SBR1031* |  | *SBR1031* |  |  |  | *SBR1031* | *SBR1031* |  |  | *SBR1031* | *SBR1031* | *SBR1031* |
| *128* | *Thermoactinomycetaceae* | *Thermoactinomycetaceae* |  | *Thermoactinomycetaceae* |  | *Thermoactinomycetaceae* | *Thermoactinomycetaceae* | *Thermoactinomycetaceae* |  | *Thermoactinomycetaceae* | *Thermoactinomycetaceae* | *Thermoactinomycetaceae* |  |
| *129* |  |  |  | *Micromonosporaceae* | *Micromonosporaceae* | *Micromonosporaceae* |  | *Micromonosporaceae* | *Micromonosporaceae* | *Micromonosporaceae* |  | *Micromonosporaceae* | *Micromonosporaceae* |
| *130* |  | *Streptomyces_thermoautotrophicus* |  | *Streptomyces_thermoautotrophicus* |  | *Streptomyces_thermoautotrophicus* |  | *Streptomyces_thermoautotrophicus* |  | *Streptomyces_thermoautotrophicus* |  | *Streptomyces_thermoautotrophicus* |  |
| *131* | *Bifidobacterium* |  | *Bifidobacterium* | *Bifidobacterium* | *Bifidobacterium* | *Bifidobacterium* | *Bifidobacterium* | *Bifidobacterium* | *Bifidobacterium* | *Bifidobacterium* | *Bifidobacterium* | *Bifidobacterium* | *Bifidobacterium* |
| *132* | *Limnobacter* |  | *Limnobacter* |  |  |  | *Limnobacter* | *Limnobacter* |  | *Limnobacter* | *Limnobacter* |  | *Limnobacter* |
| *133* |  |  |  | *Aeromicrobium* | *Aeromicrobium* | *Aeromicrobium* |  | *Aeromicrobium* |  | *Aeromicrobium* |  | *Aeromicrobium* |  |
| *134* |  | *Aminobacter* |  | *Aminobacter* |  |  |  | *Aminobacter* |  |  |  | *Aminobacter* |  |
| *135* | *Streptococcus* |  |  | *Streptococcus* |  |  |  |  | *Streptococcus* |  | *Streptococcus* |  |  |
| *136* | *Comamonadaceae2* |  |  |  |  |  |  |  |  | *Comamonadaceae2* | *Comamonadaceae2* | *Comamonadaceae2* | *Comamonadaceae2* |
| *137* | *RBG-13-54-9* |  | *RBG-13-54-9* |  |  |  | *RBG-13-54-9* |  |  | *RBG-13-54-9* | *RBG-13-54-9* |  | *RBG-13-54-9* |
| *138* | *LWQ8* |  | *LWQ8* |  | *LWQ8* | *LWQ8* | *LWQ8* | *LWQ8* | *LWQ8* |  | *LWQ8* |  | *LWQ8* |
| *139* | *Ellin6067* |  | *Ellin6067* |  |  | *Ellin6067* |  |  |  |  | *Ellin6067* |  | *Ellin6067* |
| *140* |  |  |  | *Quadrisphaera* |  | *Quadrisphaera* |  | *Quadrisphaera* |  | *Quadrisphaera* |  | *Quadrisphaera* |  |
| *141* | *PLTA13* |  |  |  |  |  | *PLTA13* |  |  | *PLTA13* | *PLTA13* |  | *PLTA13* |
| *142* | *Microtrichaceae* |  |  | *Microtrichaceae* |  | *Microtrichaceae* |  | *Microtrichaceae* |  |  | *Microtrichaceae* | *Microtrichaceae* | *Microtrichaceae* |
| *143* | *TM7a* | *TM7a* | *TM7a* |  |  |  |  | *TM7a* |  |  | *TM7a* |  | *TM7a* |
| *144* | *CL500-29_marine_group* |  | *CL500-29_marine_group* |  |  |  | *CL500-29_marine_group* |  |  | *CL500-29_marine_group* | *CL500-29_marine_group* | *CL500-29_marine_group* | *CL500-29_marine_group* |
| *145* | *Psychrobacter* |  | *Psychrobacter* |  |  |  |  |  |  |  |  | *Psychrobacter* |  |
| *146* | *Longivirga* |  | *Longivirga* |  |  |  | *Longivirga* |  |  | *Longivirga* | *Longivirga* |  | *Longivirga* |
| *147* | *Terrimonas* |  |  |  |  |  |  |  |  | *Terrimonas* | *Terrimonas* |  |  |
| *148* |  | *Craurococcus-Caldovatus* |  | *Craurococcus-Caldovatus* |  | *Craurococcus-Caldovatus* |  | *Craurococcus-Caldovatus* |  | *Craurococcus-Caldovatus* |  | *Craurococcus-Caldovatus* |  |
| *149* | *Thermomonas* |  |  |  |  |  |  |  |  |  | *Thermomonas* |  |  |
| *150* | *Gemmatimonadaceae* |  |  |  |  |  | *Gemmatimonadaceae* |  |  |  | *Gemmatimonadaceae* |  |  |
| *151* | *Pseudomonas* |  |  |  |  |  | *Pseudomonas* |  | *Pseudomonas* | *Pseudomonas* | *Pseudomonas* | *Pseudomonas* | *Pseudomonas* |
| *152* | *Propionibacteriaceae2* |  | *Propionibacteriaceae2* |  | *Propionibacteriaceae2* | *Propionibacteriaceae2* |  |  |  |  | *Propionibacteriaceae2* | *Propionibacteriaceae2* |  |
| *153* | *Sedimentibacter* |  |  |  |  |  |  |  |  |  | *Sedimentibacter* | *Sedimentibacter* |  |
| *154* | *Sphingomonadaceae1* |  |  |  |  |  |  |  | *Sphingomonadaceae1* |  | *Sphingomonadaceae1* |  | *Sphingomonadaceae1* |
| *155* | *Eubacteriaceae* | *Eubacteriaceae* | *Eubacteriaceae* |  | *Eubacteriaceae* |  | *Eubacteriaceae* | *Eubacteriaceae* |  |  | *Eubacteriaceae* |  | *Eubacteriaceae* |
| *156* |  |  |  | *Microtrichales1* |  | *Microtrichales1* |  | *Microtrichales1* |  | *Microtrichales1* | *Microtrichales1* | *Microtrichales1* |  |
| *157* | *Bacteria* | *Bacteria* | *Bacteria* | *Bacteria* | *Bacteria* | *Bacteria* | *Bacteria* | *Bacteria* | *Bacteria* | *Bacteria* | *Bacteria* |  | *Bacteria* |
| *158* | *Devosia* |  |  |  |  |  |  |  |  |  | *Devosia* |  |  |
| *159* | *Defluviicoccus* |  | *Defluviicoccus* |  |  |  |  |  | *Defluviicoccus* |  | *Defluviicoccus* | *Defluviicoccus* | *Defluviicoccus* |
| *160* | *Ferruginibacter* |  |  |  |  |  |  |  |  |  | *Ferruginibacter* |  | *Ferruginibacter* |
| *161* | *Candidatus_Berkiella* |  | *Candidatus_Berkiella* |  | *Candidatus_Berkiella* |  | *Candidatus_Berkiella* |  | *Candidatus_Berkiella* |  | *Candidatus_Berkiella* |  |  |
| *162* | *Timonella* |  |  |  |  |  |  |  |  |  | *Timonella* |  |  |
| *163* | *Kocuria* | *Kocuria* | *Kocuria* |  | *Kocuria* |  | *Kocuria* |  |  |  | *Kocuria* | *Kocuria* |  |
| *164* | *Ahniella* |  |  |  |  | *Ahniella* |  |  |  |  | *Ahniella* |  | *Ahniella* |
| *165* | *Rhodoplanes* | *Rhodoplanes* | *Rhodoplanes* |  | *Rhodoplanes* |  | *Rhodoplanes* |  |  | *Rhodoplanes* | *Rhodoplanes* | *Rhodoplanes* | *Rhodoplanes* |
| *166* |  |  | *Halobacteroidaceae* |  |  |  |  |  |  | *Halobacteroidaceae* | *Halobacteroidaceae* | *Halobacteroidaceae* | *Halobacteroidaceae* |
| *167* | *Gitt-GS-136* |  | *Gitt-GS-136* | *Gitt-GS-136* |  |  | *Gitt-GS-136* | *Gitt-GS-136* | *Gitt-GS-136* | *Gitt-GS-136* | *Gitt-GS-136* |  | *Gitt-GS-136* |
| *168* |  | *Hazenella* |  | *Hazenella* |  | *Hazenella* |  | *Hazenella* | *Hazenella* | *Hazenella* |  | *Hazenella* |  |
| *169* | *Run-SP154* |  | *Run-SP154* |  |  |  |  |  |  |  | *Run-SP154* |  |  |
| *170* | *SJA-15* |  | *SJA-15* |  |  |  |  |  | *SJA-15* | *SJA-15* | *SJA-15* | *SJA-15* |  |
| *171* | *Rhizobiaceae* | *Rhizobiaceae* | *Rhizobiaceae* |  |  |  |  | *Rhizobiaceae* | *Rhizobiaceae* |  | *Rhizobiaceae* | *Rhizobiaceae* |  |
| *172* | *Beijerinckiaceae* |  | *Beijerinckiaceae* |  | *Beijerinckiaceae* | *Beijerinckiaceae* | *Beijerinckiaceae* | *Beijerinckiaceae* | *Beijerinckiaceae* |  | *Beijerinckiaceae* | *Beijerinckiaceae* | *Beijerinckiaceae* |
| *173* |  | *Laceyella* |  |  | *Laceyella* |  |  |  |  | *Laceyella* | *Laceyella* | *Laceyella* |  |
| *174* | *966-1* |  | *966-1* |  |  |  |  | *966-1* |  |  | *966-1* | *966-1* |  |
| *175* | *Bauldia* |  | *Bauldia* |  |  |  | *Bauldia* | *Bauldia* | *Bauldia* |  | *Bauldia* | *Bauldia* |  |
| *176* |  |  |  | *Puia* |  | *Puia* |  | *Puia* |  |  |  | *Puia* |  |
| *177* | *Lachnospiraceae* |  | *Lachnospiraceae* |  |  |  |  |  |  |  |  | *Lachnospiraceae* |  |
| *178* | *Bacteroidetes_vadinHA17* |  |  |  |  |  |  |  |  |  | *Bacteroidetes_vadinHA17* |  |  |
| *179* | *Saccharimonadales1* |  |  |  |  |  |  |  | *Saccharimonadales1* |  | *Saccharimonadales1* |  | *Saccharimonadales1* |
| *180* | *Solirubrobacter* |  | *Solirubrobacter* |  |  |  | *Solirubrobacter* |  | *Solirubrobacter* |  | *Solirubrobacter* | *Solirubrobacter* | *Solirubrobacter* |
| *181* | *SWB02* |  |  |  |  |  |  |  | *SWB02* |  | *SWB02* |  | *SWB02* |
| *182* | *Pseudorhodoplanes* |  | *Pseudorhodoplanes* | *Pseudorhodoplanes* | *Pseudorhodoplanes* |  | *Pseudorhodoplanes* | *Pseudorhodoplanes* | *Pseudorhodoplanes* |  | *Pseudorhodoplanes* |  |  |
| *183* | *Dermatophilaceae2* |  | *Dermatophilaceae2* |  | *Dermatophilaceae2* |  |  |  |  |  | *Dermatophilaceae2* |  | *Dermatophilaceae2* |
| *184* | *JGI_0001001-H03* |  | *JGI_0001001-H03* |  |  | *JGI_0001001-H03* | *JGI_0001001-H03* |  |  |  | *JGI_0001001-H03* |  |  |
| *185* |  | *Chelatococcus* |  | *Chelatococcus* | *Chelatococcus* | *Chelatococcus* |  | *Chelatococcus* |  | *Chelatococcus* |  | *Chelatococcus* |  |
| *186* |  |  |  | *Thermocrispum* |  | *Thermocrispum* |  | *Thermocrispum* |  | *Thermocrispum* |  | *Thermocrispum* |  |
| *187* | *Collinsella* |  | *Collinsella* |  | *Collinsella* | *Collinsella* | *Collinsella* | *Collinsella* | *Collinsella* |  | *Collinsella* | *Collinsella* | *Collinsella* |
| *188* | *Shewanella* |  |  |  |  |  |  |  |  |  |  |  |  |
| *189* | *Ruminococcus* | *Ruminococcus* | *Ruminococcus* | *Ruminococcus* | *Ruminococcus* |  | *Ruminococcus* | *Ruminococcus* | *Ruminococcus* | *Ruminococcus* | *Ruminococcus* | *Ruminococcus* | *Ruminococcus* |
| *190* | *Actinomyces* |  | *Actinomyces* |  |  |  | *Actinomyces* |  | *Actinomyces* |  | *Actinomyces* |  | *Actinomyces* |
| *191* | *Thauera* |  |  |  |  |  | *Thauera* |  |  | *Thauera* | *Thauera* |  | *Thauera* |
| *192* | *Xanthobacteraceae* | *Xanthobacteraceae* | *Xanthobacteraceae* | *Xanthobacteraceae* | *Xanthobacteraceae* |  | *Xanthobacteraceae* |  |  |  | *Xanthobacteraceae* |  | *Xanthobacteraceae* |
| *193* | *BD1-7_clade* |  |  |  |  |  |  |  |  |  | *BD1-7_clade* | *BD1-7_clade* |  |
| *194* |  |  |  |  |  | *Sinibacillus* |  | *Sinibacillus* | *Sinibacillus* | *Sinibacillus* |  | *Sinibacillus* |  |
| *195* | *Mesorhizobium* |  | *Mesorhizobium* |  |  |  |  |  |  | *Mesorhizobium* | *Mesorhizobium* | *Mesorhizobium* | *Mesorhizobium* |
| *196* |  | *Cohnella* | *Cohnella* |  | *Cohnella* |  |  |  | *Cohnella* |  |  |  |  |
| *197* |  |  |  |  |  |  |  |  |  | *Shimazuella* |  | *Shimazuella* |  |
| *198* | *C10-SB1A* |  |  |  |  |  |  |  |  |  | *C10-SB1A* |  |  |
| *199* | *TK10* |  | *TK10* | *TK10* |  |  | *TK10* |  | *TK10* |  | *TK10* |  |  |
| *200* | *Dadabacteriales* |  | *Dadabacteriales* |  |  |  | *Dadabacteriales* |  |  |  | *Dadabacteriales* |  |  |
| *201* | *Dojkabacteria* |  | *Dojkabacteria* |  |  |  |  | *Dojkabacteria* | *Dojkabacteria* |  | *Dojkabacteria* |  | *Dojkabacteria* |
| *202* |  | *Thalassobaculales* |  | *Thalassobaculales* |  | *Thalassobaculales* |  | *Thalassobaculales* |  | *Thalassobaculales* |  |  |  |
| *203* | *OLB14* |  | *OLB14* |  |  | *OLB14* |  |  |  |  | *OLB14* |  | *OLB14* |
| *204* |  |  |  | *Pigmentiphaga* |  | *Pigmentiphaga* |  | *Pigmentiphaga* |  | *Pigmentiphaga* |  | *Pigmentiphaga* |  |
| *205* | *Neochlamydia* |  | *Neochlamydia* |  | *Neochlamydia* |  | *Neochlamydia* |  | *Neochlamydia* |  | *Neochlamydia* |  |  |
| *206* | *Pedomicrobium* |  | *Pedomicrobium* | *Pedomicrobium* | *Pedomicrobium* | *Pedomicrobium* |  | *Pedomicrobium* | *Pedomicrobium* |  | *Pedomicrobium* | *Pedomicrobium* |  |
| *207* |  |  | *Jeotgalicoccus* |  |  |  |  |  |  |  |  | *Jeotgalicoccus* |  |
| *208* | *Bacteroides* |  |  |  |  |  |  |  |  |  |  |  |  |
| *209* | *Paracoccus* | *Paracoccus* | *Paracoccus* |  |  |  |  | *Paracoccus* | *Paracoccus* | *Paracoccus* | *Paracoccus* |  | *Paracoccus* |
| *210* | *DEV007* |  | *DEV007* |  |  |  |  |  |  |  | *DEV007* |  |  |
| *211* | *Christensenellaceae_R-7_group* |  |  |  |  |  |  |  |  |  | *Christensenellaceae_R-7_group* |  |  |
| *212* | *Cellulomonas* | *Cellulomonas* |  | *Cellulomonas* | *Cellulomonas* |  |  | *Cellulomonas* |  |  | *Cellulomonas* |  |  |
| *213* | *Anaerolineaceae2* |  |  |  |  |  |  |  | *Anaerolineaceae2* |  | *Anaerolineaceae2* |  | *Anaerolineaceae2* |
| *214* | *Desulfallas-Sporotomaculum* |  | *Desulfallas-Sporotomaculum* | *Desulfallas-Sporotomaculum* |  |  |  |  | *Desulfallas-Sporotomaculum* | *Desulfallas-Sporotomaculum* | *Desulfallas-Sporotomaculum* | *Desulfallas-Sporotomaculum* | *Desulfallas-Sporotomaculum* |
| *215* | *Atopobium* |  | *Atopobium* | *Atopobium* |  | *Atopobium* | *Atopobium* | *Atopobium* | *Atopobium* |  | *Atopobium* |  | *Atopobium* |
| *216* | *Candidatus_Alysiosphaera* |  |  |  |  |  |  |  |  |  | *Candidatus_Alysiosphaera* |  |  |
| *217* | *Rhizobiales* |  |  |  |  | *Rhizobiales* | *Rhizobiales* |  |  |  | *Rhizobiales* | *Rhizobiales* |  |
| *218* | *Arenicellaceae* |  | *Arenicellaceae* |  |  |  |  |  | *Arenicellaceae* |  | *Arenicellaceae* |  |  |
| *219* | *Subgroup_10* |  |  |  |  |  | *Subgroup_10* |  |  |  | *Subgroup_10* |  | *Subgroup_10* |
| *220* | *Novosphingobium* |  |  |  |  |  |  |  |  |  | *Novosphingobium* |  | *Novosphingobium* |
| *221* | *Gallicola* |  | *Gallicola* |  |  |  |  |  |  |  | *Gallicola* | *Gallicola* |  |
| *222* | *JCM_18997* |  | *JCM_18997* | *JCM_18997* |  |  |  | *JCM_18997* |  |  | *JCM_18997* |  |  |
| *223* | *Truepera* |  | *Truepera* | *Truepera* |  | *Truepera* |  | *Truepera* |  | *Truepera* | *Truepera* | *Truepera* | *Truepera* |
| *224* |  |  |  | *Filomicrobium* |  | *Filomicrobium* |  | *Filomicrobium* |  | *Filomicrobium* |  | *Filomicrobium* |  |
| *225* | *Subgroup_7* |  |  |  |  |  |  |  |  |  | *Subgroup_7* |  |  |
| *226* | *SJA-28* |  | *SJA-28* |  | *SJA-28* |  | *SJA-28* |  | *SJA-28* |  | *SJA-28* | *SJA-28* | *SJA-28* |
| *227* |  |  |  |  |  | *Ammoniibacillus* |  |  |  | *Ammoniibacillus* |  | *Ammoniibacillus* |  |
| *228* | *Thiobacillus* |  |  |  |  |  |  |  |  |  | *Thiobacillus* |  |  |
| *229* |  | *Brevibacterium* |  |  |  |  |  |  |  |  |  |  |  |
| *230* |  |  | *Burkholderia-Caballeronia-Paraburkholderia* |  |  | *Burkholderia-Caballeronia-Paraburkholderia* |  |  |  | *Burkholderia-Caballeronia-Paraburkholderia* |  | *Burkholderia-Caballeronia-Paraburkholderia* |  |
| *231* |  | *Tepidimicrobium* | *Tepidimicrobium* | *Tepidimicrobium* |  |  |  | *Tepidimicrobium* | *Tepidimicrobium* | *Tepidimicrobium* |  | *Tepidimicrobium* | *Tepidimicrobium* |
| *232* | *Gemmataceae* |  | *Gemmataceae* |  |  | *Gemmataceae* | *Gemmataceae* |  |  | *Gemmataceae* | *Gemmataceae* | *Gemmataceae* |  |
| *233* | *Pseudonocardia* |  | *Pseudonocardia* |  | *Pseudonocardia* |  | *Pseudonocardia* | *Pseudonocardia* |  |  | *Pseudonocardia* |  |  |
| *234* |  | *Pedobacter* | *Pedobacter* | *Pedobacter* |  |  |  |  |  |  |  |  |  |
| *235* | *Chitinophagales2* |  |  |  |  |  |  |  |  |  | *Chitinophagales2* |  |  |
| *236* | *RsaHf231* |  |  |  |  |  |  |  |  |  | *RsaHf231* |  |  |
| *237* | *Patescibacteria* |  | *Patescibacteria* | *Patescibacteria* | *Patescibacteria* | *Patescibacteria* | *Patescibacteria* |  | *Patescibacteria* |  | *Patescibacteria* | *Patescibacteria* | *Patescibacteria* |
| *238* | *DS-100* |  | *DS-100* |  |  | *DS-100* |  |  |  |  | *DS-100* |  |  |
| *239* | *Sandaracinaceae* | *Sandaracinaceae* | *Sandaracinaceae* |  |  | *Sandaracinaceae* |  |  | *Sandaracinaceae* |  | *Sandaracinaceae* | *Sandaracinaceae* | *Sandaracinaceae* |
| *240* | *A0839* |  |  |  |  | *A0839* |  |  |  |  | *A0839* |  | *A0839* |
| *241* | *unclassified_p__Patescibacteria* |  |  |  |  |  | *unclassified_p__Patescibacteria* |  |  | *unclassified_p__Patescibacteria* | *unclassified_p__Patescibacteria* | *unclassified_p__Patescibacteria* | *unclassified_p__Patescibacteria* |
| *242* | *Comamonadaceae1* |  |  |  |  |  |  |  |  |  | *Comamonadaceae1* |  | *Comamonadaceae1* |
| *243* | *Candidatus_Caldatribacterium* | *Candidatus_Caldatribacterium* | *Candidatus_Caldatribacterium* |  |  |  |  |  |  |  | *Candidatus_Caldatribacterium* |  | *Candidatus_Caldatribacterium* |
| *244* | *Eubacterium_coprostanoligenes_group* |  |  |  |  |  |  |  |  |  |  |  |  |
| *245* | *WWH38* |  |  |  |  |  | *WWH38* |  |  |  | *WWH38* |  |  |
| *246* | *Lautropia* |  |  |  |  |  |  |  | *Lautropia* |  | *Lautropia* |  | *Lautropia* |
| *247* | *Longilinea* |  |  |  |  |  |  |  |  |  | *Longilinea* |  |  |
| *248* | *Anaerolinea* |  |  |  |  |  |  |  |  |  | *Anaerolinea* |  | *Anaerolinea* |
| *249* | *Camelimonas* |  |  |  |  |  | *Camelimonas* |  | *Camelimonas* |  | *Camelimonas* |  |  |
| *250* |  | *Alphaproteobacteria2* | *Alphaproteobacteria2* |  |  | *Alphaproteobacteria2* |  | *Alphaproteobacteria2* |  |  | *Alphaproteobacteria2* |  |  |
| *251* |  |  | *Hungateiclostridiaceae1* |  | *Hungateiclostridiaceae1* |  |  | *Hungateiclostridiaceae1* |  | *Hungateiclostridiaceae1* | *Hungateiclostridiaceae1* | *Hungateiclostridiaceae1* | *Hungateiclostridiaceae1* |
| *252* | *Acidobacteriales* |  | *Acidobacteriales* | *Acidobacteriales* |  |  |  | *Acidobacteriales* |  |  | *Acidobacteriales* |  | *Acidobacteriales* |
| *253* | *Stenotrophobacter* |  |  |  |  |  |  |  |  |  | *Stenotrophobacter* | *Stenotrophobacter* | *Stenotrophobacter* |
| *254* |  |  |  |  |  |  | *Chryseobacterium* | *Chryseobacterium* |  |  |  | *Chryseobacterium* | *Chryseobacterium* |
| *255* | *Labrys* |  |  |  |  |  |  |  |  |  | *Labrys* |  |  |
| *256* | *Reyranella* |  |  |  |  |  |  |  |  |  | *Reyranella* |  |  |
| *257* | *Synergistaceae* |  |  |  |  |  |  |  | *Synergistaceae* |  | *Synergistaceae* |  |  |
| *258* |  |  |  |  | *Alkaliphilus* | *Alkaliphilus* |  | *Alkaliphilus* | *Alkaliphilus* | *Alkaliphilus* |  | *Alkaliphilus* |  |
| *259* |  | *Nonomuraea* |  | *Nonomuraea* |  | *Nonomuraea* |  | *Nonomuraea* | *Nonomuraea* | *Nonomuraea* | *Nonomuraea* | *Nonomuraea* |  |
| *260* | *Hyphomicrobiaceae2* |  | *Hyphomicrobiaceae2* |  |  | *Hyphomicrobiaceae2* |  |  |  |  | *Hyphomicrobiaceae2* |  |  |
| *261* |  | *Peptostreptococcaceae* |  |  | *Peptostreptococcaceae* | *Peptostreptococcaceae* |  | *Peptostreptococcaceae* | *Peptostreptococcaceae* |  |  |  |  |
| *262* | *Propionibacterium* |  |  |  |  |  |  |  |  |  |  |  |  |
| *263* | *Roseomonas* |  | *Roseomonas* |  |  |  |  | *Roseomonas* |  |  | *Roseomonas* |  |  |
| *264* | *Rubellimicrobium* |  | *Rubellimicrobium* |  |  |  |  |  |  |  | *Rubellimicrobium* |  |  |
| *265* | *Nitrospira* |  |  |  |  | *Nitrospira* | *Nitrospira* |  |  |  | *Nitrospira* |  |  |
| *266* | *Parcubacteria* |  | *Parcubacteria* |  |  |  |  |  |  |  | *Parcubacteria* |  | *Parcubacteria* |
| *267* | *Vermiphilaceae* |  | *Vermiphilaceae* |  |  |  |  |  | *Vermiphilaceae* |  |  |  |  |
| *268* | *SAR324_cladeMarine_group_B* |  | *SAR324_cladeMarine_group_B* |  |  |  |  |  | *SAR324_cladeMarine_group_B* |  | *SAR324_cladeMarine_group_B* |  |  |
| *269* | *Bosea* |  | *Bosea* |  |  |  | *Bosea* |  |  |  | *Bosea* | *Bosea* |  |
| *270* | *Gemmobacter* |  | *Gemmobacter* |  |  |  |  |  |  |  | *Gemmobacter* |  |  |
| *271* | *OM190* |  |  |  |  |  |  |  |  |  | *OM190* |  |  |
| *272* |  |  | *WWE3* |  |  |  |  |  |  |  | *WWE3* | *WWE3* |  |
| *273* | *Fimbriimonadaceae* |  | *Fimbriimonadaceae* |  |  |  |  |  |  |  | *Fimbriimonadaceae* |  |  |
| *274* |  |  |  | *Sphaerobacter* |  | *Sphaerobacter* |  | *Sphaerobacter* |  | *Sphaerobacter* |  |  |  |
| *275* |  |  |  |  |  | *unclassified_p__Firmicutes* |  | *unclassified_p__Firmicutes* |  | *unclassified_p__Firmicutes* |  | *unclassified_p__Firmicutes* |  |
| *276* | *AKYH767* |  |  |  |  |  |  |  |  |  |  |  |  |
| *277* | *Eubacterium_brachy_group* |  | *Eubacterium_brachy_group* |  |  |  |  |  |  |  | *Eubacterium_brachy_group* |  |  |
| *278* | *Reyranellaceae* |  |  |  |  |  |  |  | *Reyranellaceae* |  | *Reyranellaceae* |  |  |
| *279* | *NS9_marine_group* |  |  |  |  |  |  |  |  |  | *NS9_marine_group* |  |  |
| *280* | *Actinomarinales* |  | *Actinomarinales* |  | *Actinomarinales* |  | *Actinomarinales* |  |  | *Actinomarinales* | *Actinomarinales* |  |  |
| *281* | *Bdellovibrio* |  | *Bdellovibrio* |  |  |  |  |  | *Bdellovibrio* | *Bdellovibrio* | *Bdellovibrio* |  |  |
| *282* |  |  |  |  |  | *Longispora* |  | *Longispora* |  | *Longispora* |  | *Longispora* |  |
| *283* |  |  |  |  |  |  |  |  |  | *Tumebacillus* |  | *Tumebacillus* |  |
| *284* | *Verrucomicrobiaceae1* |  | *Verrucomicrobiaceae1* |  |  |  |  |  | *Verrucomicrobiaceae1* |  | *Verrucomicrobiaceae1* |  |  |
| *285* |  |  | *TM7x* |  |  |  | *TM7x* |  |  |  | *TM7x* |  | *TM7x* |
| *286* | *Haliangium* |  |  |  |  |  |  |  |  |  | *Haliangium* |  |  |
| *287* | *Amb-16S-1323* |  | *Amb-16S-1323* |  | *Amb-16S-1323* |  | *Amb-16S-1323* |  |  |  | *Amb-16S-1323* |  |  |
| *288* | *Microlunatus* |  | *Microlunatus* |  | *Microlunatus* |  |  |  |  |  | *Microlunatus* |  |  |
| *289* |  |  |  | *Lutispora* | *Lutispora* |  |  |  |  | *Lutispora* | *Lutispora* | *Lutispora* | *Lutispora* |
| *290* |  |  |  |  |  |  |  |  |  |  | *Sphingosinicella* |  | *Sphingosinicella* |
| *291* | *Candidatus_Competibacter* |  |  |  |  |  |  |  |  |  | *Candidatus_Competibacter* | *Candidatus_Competibacter* | *Candidatus_Competibacter* |
| *292* | *Dermatophilaceae1* |  | *Dermatophilaceae1* |  |  |  |  |  |  |  | *Dermatophilaceae1* |  |  |
| *293* | *Ilumatobacteraceae1* |  |  |  |  |  |  |  |  |  | *Ilumatobacteraceae1* |  |  |
| *294* | *Chlamydiales* |  | *Chlamydiales* |  |  |  | *Chlamydiales* |  | *Chlamydiales* |  | *Chlamydiales* |  |  |
| *295* | *Olsenella* |  | *Olsenella* | *Olsenella* |  |  | *Olsenella* |  |  |  | *Olsenella* |  |  |
| *296* | *Sporacetigenium* |  |  |  | *Sporacetigenium* |  |  |  | *Sporacetigenium* |  | *Sporacetigenium* | *Sporacetigenium* |  |
| *297* | *Flavobacterium* | *Flavobacterium* |  |  |  |  | *Flavobacterium* |  |  |  | *Flavobacterium* |  |  |
| *298* | *Parachlamydiaceae* |  | *Parachlamydiaceae* |  | *Parachlamydiaceae* |  |  |  |  |  |  |  |  |
| *299* | *Aquisphaera* |  | *Aquisphaera* |  |  |  | *Aquisphaera* |  |  | *Aquisphaera* | *Aquisphaera* |  |  |
| *300* |  |  |  |  |  |  | *Clostridium_sensu_stricto_3* |  |  | *Clostridium_sensu_stricto_3* | *Clostridium_sensu_stricto_3* | *Clostridium_sensu_stricto_3* | *Clostridium_sensu_stricto_3* |
| *301* |  |  |  |  |  |  |  |  | *Gracilibacter* |  | *Gracilibacter* | *Gracilibacter* | *Gracilibacter* |
| *302* |  |  |  |  |  |  | *S085* |  |  |  | *S085* |  |  |
| *303* | *Chitinophagales1* |  |  |  |  |  |  |  |  |  |  |  |  |
| *304* | *Flaviflexus* |  |  |  |  |  |  |  |  |  |  |  |  |
| *305* | *Anaerovorax* |  |  |  |  |  |  |  |  |  | *Anaerovorax* |  | *Anaerovorax* |
| *306* | *alphaI_cluster* |  | *alphaI_cluster* |  |  |  |  |  |  |  | *alphaI_cluster* |  |  |
| *307* | *Acidovorax* |  |  |  |  |  | *Acidovorax* |  |  |  | *Acidovorax* |  |  |
| *308* | *OLB13* |  |  |  |  |  | *OLB13* |  |  |  | *OLB13* | *OLB13* |  |
| *309* | *Rhodobacteraceae* |  | *Rhodobacteraceae* |  |  |  | *Rhodobacteraceae* |  |  |  | *Rhodobacteraceae* | *Rhodobacteraceae* |  |
| *310* | *Roseburia* |  | *Roseburia* |  | *Roseburia* |  | *Roseburia* |  | *Roseburia* |  | *Roseburia* | *Roseburia* | *Roseburia* |
| *311* | *Sporichthyaceae* |  |  |  |  |  |  |  |  | *Sporichthyaceae* | *Sporichthyaceae* |  | *Sporichthyaceae* |
| *312* | *Stella* |  |  |  |  |  |  |  |  |  | *Stella* |  |  |
| *313* | *Subgroup_22* |  |  |  |  |  |  |  |  |  | *Subgroup_22* |  | *Subgroup_22* |
| *314* |  |  |  |  |  |  | *Alcaligenaceae* | *Alcaligenaceae* | *Alcaligenaceae* |  |  |  |  |
| *315* |  |  | *Alphaproteobacteria1* |  |  |  |  | *Alphaproteobacteria1* |  |  | *Alphaproteobacteria1* |  | *Alphaproteobacteria1* |
| *316* | *Acetobacterium* |  |  |  |  |  |  |  |  |  | *Acetobacterium* |  |  |
| *317* | *Phaeodactylibacter* |  |  |  |  |  |  |  |  |  | *Phaeodactylibacter* |  |  |
| *318* | *AKYG587* |  |  |  |  |  |  |  |  |  | *AKYG587* |  |  |
| *319* | *Candidatus_Curtissbacteria* |  |  |  |  |  |  |  |  |  | *Candidatus_Curtissbacteria* |  |  |
| *320* | *Dokdonella* |  |  |  |  |  |  |  |  | *Dokdonella* | *Dokdonella* |  |  |
| *321* | *Nakamurella* |  |  |  |  |  |  |  |  |  | *Nakamurella* |  |  |
| *322* | *Serratia* |  |  |  |  |  |  |  | *Serratia* |  | *Serratia* |  |  |
| *323* | *Waddlia* |  |  |  |  |  |  |  |  |  | *Waddlia* |  |  |
| *324* | *Candidatus_Kerfeldbacteria* |  | *Candidatus_Kerfeldbacteria* |  |  |  |  |  |  |  | *Candidatus_Kerfeldbacteria* |  |  |
| *325* |  |  | *Babeliaceae* |  |  |  |  | *Babeliaceae* | *Babeliaceae* | *Babeliaceae* | *Babeliaceae* |  |  |
| *326* |  |  | *Holosporaceae* |  |  |  |  |  | *Holosporaceae* |  | *Holosporaceae* |  |  |
| *327* |  |  |  |  |  | *Pantoea* |  |  |  |  | *Pantoea* |  |  |
| *328* |  |  | *Solibacillus* |  |  |  |  |  | *Solibacillus* | *Solibacillus* | *Solibacillus* | *Solibacillus* |  |
| *329* |  |  | *Solirubrobacteraceae* |  |  |  | *Solirubrobacteraceae* |  |  | *Solirubrobacteraceae* | *Solirubrobacteraceae* |  | *Solirubrobacteraceae* |
| *330* |  |  |  |  |  |  |  |  |  |  | *Verrucomicrobiaceae2* |  |  |
| *331* | *Babeliales1* |  | *Babeliales1* |  |  |  |  |  |  |  |  |  |  |
| *332* | *PHOS-HE36* |  |  |  |  |  |  |  |  |  | *PHOS-HE36* | *PHOS-HE36* |  |
| *333* | *SM2D12* |  |  |  |  |  |  |  |  |  | *SM2D12* |  |  |
| *334* |  |  |  |  |  |  |  |  |  |  | *Acidibacter* |  | *Acidibacter* |
| *335* |  |  |  |  |  |  |  |  |  |  | *Methyloversatilis* |  |  |
| *336* |  |  |  |  |  |  |  |  |  |  |  |  | *Parvibaculum* |
| *337* |  |  |  |  |  |  |  | *Stenotrophomonas* | *Stenotrophomonas* |  |  | *Stenotrophomonas* |  |
| *338* |  |  |  |  |  |  | *Syntrophaceticus* |  |  |  | *Syntrophaceticus* | *Syntrophaceticus* |  |
| *339* |  |  |  |  |  |  | *Thermomicrobiaceae* |  | *Thermomicrobiaceae* | *Thermomicrobiaceae* | *Thermomicrobiaceae* |  |  |
| *340* |  |  |  | *Thermopolyspora* |  | *Thermopolyspora* |  | *Thermopolyspora* | *Thermopolyspora* |  | *Thermopolyspora* |  |  |
| *341* | *Beutenbergiaceae* |  |  |  |  |  |  |  |  |  |  |  |  |
| *342* | *Dysgonomonas* |  |  |  |  |  |  |  |  |  |  |  |  |
| *343* | *Simkaniaceae* |  |  |  |  |  |  |  |  |  |  |  |  |
| *344* | *Candidatus_Paracaedibacter* |  |  |  |  |  |  |  |  |  | *Candidatus_Paracaedibacter* |  |  |
| *345* | *Desulfobacterota* |  |  |  |  |  |  |  |  |  | *Desulfobacterota* |  |  |
| *346* | *NB1-j* |  |  |  |  |  |  |  |  |  | *NB1-j* |  |  |
| *347* |  |  |  | *Hahella* |  |  |  | *Hahella* |  |  |  |  |  |
| *348* |  |  | *Leptolinea* |  |  |  |  |  |  |  | *Leptolinea* |  |  |
| *349* |  |  |  |  |  |  |  | *Pseudoclostridium* | *Pseudoclostridium* | *Pseudoclostridium* |  | *Pseudoclostridium* |  |
| *350* | *Blautia* |  |  |  |  |  |  |  |  |  | *Blautia* |  |  |
| *351* | *Lactococcus* |  |  |  |  |  | *Lactococcus* |  |  |  | *Lactococcus* |  |  |
| *352* | *Acidaminococcaceae* |  |  |  |  |  |  |  |  |  | *Acidaminococcaceae* |  |  |
| *353* | *Hespellia* |  | *Hespellia* |  |  |  |  |  | *Hespellia* |  | *Hespellia* |  |  |
| *354* | *Prosthecomicrobium* |  |  |  |  |  |  |  |  | *Prosthecomicrobium* | *Prosthecomicrobium* |  |  |
| *355* | *Sphingobium* |  |  |  |  |  |  |  |  |  | *Sphingobium* |  |  |
| *356* | *Vicinamibacteraceae* |  |  |  |  |  |  | *Vicinamibacteraceae* |  |  | *Vicinamibacteraceae* | *Vicinamibacteraceae* |  |
| *357* |  | *Acetobacteraceae* |  | *Acetobacteraceae* |  | *Acetobacteraceae* |  | *Acetobacteraceae* |  |  |  |  |  |
| *358* |  |  |  |  |  |  |  |  |  |  | *CCM19a* | *CCM19a* | *CCM19a* |
| *359* |  |  |  |  |  |  |  |  |  |  | *unclassified_p__Chloroflexi* |  |  |
| *360* | *Amphiplicatus* |  |  |  |  |  |  |  |  |  | *Amphiplicatus* |  |  |
| *361* | *Arthrobacter* |  |  |  |  |  | *Arthrobacter* |  |  |  |  |  |  |
| *362* | *Anaerosalibacter* |  |  |  |  |  |  |  |  | *Anaerosalibacter* | *Anaerosalibacter* | *Anaerosalibacter* | *Anaerosalibacter* |
| *363* | *Desulfobulbus* |  |  |  |  |  |  |  |  |  | *Desulfobulbus* |  |  |
| *364* | *Paludibacter* |  |  |  |  |  |  |  |  |  | *Paludibacter* |  |  |
| *365* | *Paludisphaera* |  | *Paludisphaera* |  |  |  |  |  |  |  |  |  |  |
| *366* | *SB-5* |  |  |  |  |  |  |  |  |  | *SB-5* |  | *SB-5* |
| *367* | *Syntrophobacteraceae* |  |  |  |  |  |  |  |  |  | *Syntrophobacteraceae* |  |  |
| *368* |  |  |  | *Acetitomaculum* |  |  |  |  |  |  | *Acetitomaculum* |  | *Acetitomaculum* |
| *369* |  |  | *Acidimicrobiia2* |  |  |  |  |  |  |  | *Acidimicrobiia2* |  |  |
| *370* |  |  | *Babeliales2* |  |  |  |  |  |  |  |  |  |  |
| *371* |  |  | *Candidatus_Protochlamydia* |  |  |  |  |  |  |  | *Candidatus_Protochlamydia* |  |  |
| *372* |  |  |  |  |  |  |  |  |  |  | *Chromatiaceae* |  |  |
| *373* |  |  |  |  | *Clostridium_sensu_stricto_11* | *Clostridium_sensu_stricto_11* | *Clostridium_sensu_stricto_11* |  |  | *Clostridium_sensu_stricto_11* | *Clostridium_sensu_stricto_11* |  |  |
| *374* |  | *Cutibacterium* | *Cutibacterium* |  |  |  |  |  |  | *Cutibacterium* |  |  |  |
| *375* |  |  |  |  |  |  |  |  | *Denitratisoma* |  | *Denitratisoma* |  |  |
| *376* |  |  | *Desulfoprunum* |  |  |  | *Desulfoprunum* |  |  |  | *Desulfoprunum* |  |  |
| *377* |  |  | *Saccharimonadaceae* |  |  |  |  |  |  |  | *Saccharimonadaceae* |  |  |
| *378* |  |  |  |  |  |  |  |  |  |  | *Vicinamibacterales* |  | *Vicinamibacterales* |
| *379* | *Agromyces* |  |  |  |  |  |  |  |  |  |  |  |  |
| *380* | *Fastidiosipila* |  |  |  |  |  |  |  |  |  |  |  |  |
| *381* | *Eggerthella* |  | *Eggerthella* |  |  |  |  |  |  |  | *Eggerthella* |  |  |
| *382* | *OLB12* |  |  |  |  |  |  |  |  |  | *OLB12* |  |  |
| *383* | *Petrimonas* |  | *Petrimonas* |  |  |  |  |  |  |  | *Petrimonas* |  |  |
| *384* | *Pseudoxanthomonas* |  |  |  |  |  |  |  |  |  | *Pseudoxanthomonas* |  |  |
| *385* |  |  |  |  |  |  |  |  |  | *Anaerolineae* | *Anaerolineae* | *Anaerolineae* |  |
| *386* |  |  |  |  |  |  | *Clostridium_sensu_stricto_12* |  |  |  | *Clostridium_sensu_stricto_12* | *Clostridium_sensu_stricto_12* | *Clostridium_sensu_stricto_12* |
| *387* |  |  |  |  |  |  | *Nocardia* | *Nocardia* |  |  | *Nocardia* | *Nocardia* | *Nocardia* |
| *388* |  |  | *Pajaroellobacter* |  |  |  |  |  |  |  | *Pajaroellobacter* | *Pajaroellobacter* | *Pajaroellobacter* |
| *389* |  |  |  |  |  |  |  |  |  |  |  | *Sporosalibacterium* |  |
| *390* |  |  |  |  |  | *Symbiobacteriales* |  | *Symbiobacteriales* |  |  |  | *Symbiobacteriales* |  |
| *391* | *Anaeromyxobacter* |  |  |  |  |  |  |  |  |  | *Anaeromyxobacter* |  |  |
| *392* | *Bacteroidia* |  |  |  |  |  |  |  |  |  | *Bacteroidia* |  |  |
| *393* | *Candidatus_Ovatusbacter* |  |  |  |  |  |  |  |  |  | *Candidatus_Ovatusbacter* |  |  |
| *394* | *Senegalimassilia* |  | *Senegalimassilia* |  |  |  |  |  |  |  | *Senegalimassilia* |  |  |
| *395* |  |  |  |  |  | *Blastocatellaceae2* |  |  |  |  | *Blastocatellaceae2* |  |  |
| *396* |  |  |  |  |  |  | *Caulobacter* |  |  |  | *Caulobacter* |  |  |
| *397* |  |  |  |  | *Cellulosilyticum* |  | *Cellulosilyticum* |  |  | *Cellulosilyticum* | *Cellulosilyticum* |  |  |
| *398* |  |  | *Coriobacteriales* | *Coriobacteriales* |  |  | *Coriobacteriales* |  | *Coriobacteriales* |  | *Coriobacteriales* |  |  |
| *399* |  |  | *Eubacterium* |  |  |  |  |  | *Eubacterium* |  | *Eubacterium* |  |  |
| *400* |  |  |  |  |  | *WN-HWB-116* |  |  |  | *WN-HWB-116* |  |  |  |
| *401* | *0319-6G20* |  |  |  |  |  |  |  |  | *0319-6G20* |  |  |  |
| *402* | *Gammaproteobacteria* |  | *Gammaproteobacteria* |  |  |  |  |  |  |  |  |  |  |
| *403* | *Microscillaceae* |  |  |  |  |  |  |  |  |  | *Microscillaceae* |  |  |
| *404* | *Rikenellaceae* |  |  |  |  |  |  |  |  |  |  |  | *Rikenellaceae* |
| *405* | *Sphingomonas* |  |  |  |  |  |  |  |  |  | *Sphingomonas* |  |  |
| *406* |  |  |  |  |  |  |  |  |  |  |  | *Alicyclobacillus* |  |
| *407* |  |  |  |  |  |  | *Fusobacterium* | *Fusobacterium* |  |  | *Fusobacterium* |  |  |
| *408* |  |  |  |  |  |  |  |  |  |  | *JG30-KF-CM66* |  |  |
| *409* |  |  |  |  | *Lachnospiraceae_ND3007_group* |  |  |  |  | *Lachnospiraceae_ND3007_group* | *Lachnospiraceae_ND3007_group* |  |  |
| *410* |  |  |  |  |  |  |  |  |  |  | *Luteitalea* |  |  |
| *411* |  |  |  |  |  | *Marinococcaceae* |  |  | *Marinococcaceae* |  |  |  |  |
| *412* |  |  |  |  |  |  | *OLB15* |  |  |  | *OLB15* |  |  |
| *413* |  | *Ruminiclostridium* | *Ruminiclostridium* |  |  |  |  |  |  | *Ruminiclostridium* |  | *Ruminiclostridium* |  |
| *414* |  |  |  |  |  |  |  |  |  |  | *Ruminococcus_torques_group* |  |  |
| *415* |  |  |  |  |  | *Rummeliibacillus* |  |  |  |  |  | *Rummeliibacillus* |  |
| *416* |  |  |  |  |  |  |  |  |  | *Symbiobacterium* |  |  | *Symbiobacterium* |
| *417* |  |  |  |  |  |  |  |  |  |  |  | *Syntrophobotulus* |  |
| *418* |  |  |  |  | *Thermoactinomyces* |  |  |  |  |  |  | *Thermoactinomyces* |  |
| *419* |  |  |  |  |  |  |  |  |  |  | *Acidimicrobiia1* |  |  |
| *420* |  |  |  |  |  |  |  |  |  |  | *Actinomycetaceae2* |  |  |
| *421* |  |  |  |  |  |  | *Anaerovoracaceae* |  |  |  | *Anaerovoracaceae* |  |  |
| *422* |  |  |  |  | *CAG-352* |  | *CAG-352* |  |  | *CAG-352* |  |  |  |
| *423* |  |  |  |  |  |  |  |  |  |  | *Cloacibacterium* |  |  |
| *424* |  |  |  |  |  |  |  |  |  |  |  | *Desulfitobacterium* |  |
| *425* |  |  |  | *Hungateiclostridium* |  |  |  |  |  | *Hungateiclostridium* |  |  |  |
| *426* |  |  |  |  |  |  | *Luteococcus* | *Luteococcus* |  |  | *Luteococcus* |  |  |
| *427* |  |  | *Monoglobus* |  |  |  |  |  |  |  | *Monoglobus* |  |  |
| *428* |  |  |  |  |  |  |  |  |  |  |  | *Pelotomaculum* |  |
| *429* |  |  |  |  |  |  |  |  |  |  | *SM1A02* |  |  |
| *430* |  |  |  |  |  |  |  |  |  |  |  | *Tepidibacillus* |  |
| *431* |  |  |  |  |  |  |  |  |  |  | *Aminicenantales* |  |  |
| *432* |  |  |  |  |  |  |  |  |  |  | *BSV26* |  |  |
| *433* |  |  |  |  |  |  |  |  |  |  | *Candidatus_Woesebacteria* |  |  |
| *434* |  |  |  |  |  |  |  |  |  |  | *CI75cm.2.12* |  |  |
| *435* |  |  |  |  |  |  |  |  |  |  |  | *Comamonas* |  |
| *436* |  |  |  |  |  |  |  |  |  | *Desulfotomaculum* |  | *Desulfotomaculum* |  |
| *437* |  |  |  |  |  |  |  |  |  |  | *Desulfovibrio* |  |  |
| *438* |  |  |  |  |  |  |  |  |  |  | *Dinghuibacter* |  |  |
| *439* |  |  |  |  |  |  |  |  |  |  |  | *Elsterales* |  |
| *440* |  |  |  |  |  |  |  |  |  |  | *Hungateiclostridiaceae2* | *Hungateiclostridiaceae2* |  |
| *441* |  |  |  |  |  |  |  |  |  |  | *Legionellaceae* |  |  |
| *442* |  |  |  |  |  |  |  |  |  | *MB-A2-108* |  |  |  |
| *443* |  |  |  |  |  |  |  |  |  |  | *Methylocaldum* |  | *Methylocaldum* |
| *444* |  |  |  |  |  |  |  |  |  |  | *Methylophilaceae* |  |  |
| *445* |  |  |  |  |  |  |  |  |  | *MND1* |  | *MND1* |  |
| *446* |  |  |  |  |  |  |  |  |  |  | *Oricola* |  |  |
| *447* |  |  |  |  |  |  |  |  |  |  |  | *Oxobacter* |  |
| *448* |  |  |  |  |  |  |  |  |  |  | *Pelolinea* |  |  |
| *449* |  |  |  |  |  |  |  |  |  |  | *Prevotella* |  |  |
| *450* |  |  |  |  |  |  |  |  |  |  | *Sumerlaeia* |  |  |

(CK: ordinary sludge compost without microplastics at 55 ℃; L1: ordinary sludge compost with 10% microplastics added at 55 ℃; L2: high-temperature sludge compost with 10% microplastics added at 70 ℃)
